# Molecular Epidemiology of Respiratory Syncytial Virus and Rhinovirus in Santander, Colombia, During the COVID-19 Pandemic and Post-Pandemic Periods, 2020–2024

**DOI:** 10.3390/v18060666

**Published:** 2026-06-12

**Authors:** William Fernando Chaparro-Pico, Nathalia Bueno, Anyela Lozano-Parra, Jürg Niederbacher, Víctor Herrera, Luis Miguel Sosa Ávila, Mayra Alejandra Machuca Pérez, Martha Lucía Díaz Galvis

**Affiliations:** 1 Grupo de Inmunología y Epidemiología Molecular (GIEM), Escuela de Microbiología, Universidad Industrial de Santander UIS, Calle 9 Carrera 27, Bucaramanga 680002, Colombia; williamfernandochaparro@gmail.com (W.F.C.-P.); nathaliabuenoariza@gmail.com (N.B.); maymaper@uis.edu.co (M.A.M.P.); 2 Grupo Epidemiología Clínica, Escuela de Medicina, Universidad Industrial de Santander UIS, Calle 9 Carrera 27, Bucaramanga 680002, Colombia; anyela.lozano@correo.uis.edu.co (A.L.-P.); jurgnied@uis.edu.co (J.N.); vicmaher@uis.edu.co (V.H.); 3 Grupo de Investigación de Pediatría (PAIDÓS), Departamento de Pediatría, Universidad Industrial de Santander UIS, Calle 9 Carrera 27, Bucaramanga 680002, Colombia; lmsosavi@uis.edu.co; 4 Grupo de Investigación en Demografía, Salud Pública y Sistemas de Salud (GUINDESS), Escuela de Medicina, Universidad Industrial de Santander UIS, Calle 9 Carrera 27, Bucaramanga 680002, Colombia

**Keywords:** respiratory syncytial virus, rhinovirus, molecular epidemiology, phylogenetic analysis

## Abstract

Respiratory syncytial virus (RSV) and rhinovirus (RV) are major causes of acute respiratory infections (ARIs) worldwide. The circulation of both RSV and RV was notably altered during the COVID-19 pandemic. This study analyzed the epidemiological and molecular characteristics of RSV and RV in Santander, Colombia, during the pandemic period (PP, 2020–2021) and the post-pandemic period (PPP, 2023–2024). A total of 921 respiratory samples from patients with ARIs were screened for RSV-A/B and RV. Sequences of the RSV attachment glycoprotein (G) gene and the RV VP4–VP2 region were analyzed in positive samples. Epidemiological and clinical characteristics of the study population were also assessed. RSV was not detected during PP, whereas RV was detected in 4.8% of samples. During PPP, RSV-A, RSV-B, and RV were detected in 5.6%, 4.4%, and 28.0% of samples, respectively. RSV infections were mainly identified in children, while RV was detected across all age groups. RSV-A sequences grouped within seven A.D-derived lineages (A.D.1.5, A.D.1.7, A.D.1.8, A.D.3.2, A.D.3.3, A.D.5.1, A.D.5.2), whereas RSV-B sequences clustered within the B.D.E.1 lineage. RV showed a higher number of detected genotypes during the PPP than during the PP. The genotypic characterization performed in this study provides new insight into the molecular epidemiology of RSV and RV in Santander, Colombia, during PP and PPP, and represents, to our knowledge, the first regional description of RSV lineages and RV genotypes across these periods.

## 1. Introduction

Respiratory syncytial virus (RSV) and Rhinovirus (RV) are two pathogens able to cause acute respiratory infections (ARIs) [1], with symptoms ranging from mild to severe and even fatal disease [2,3,4,5]. RSV is the most common cause of lower respiratory tract infections in young infants and one of the main causes of severe ARIs in children and older adults [6,7,8]. It is estimated that more than 60% of children are infected with this virus during their first year of life, and nearly all children experience at least one RSV infection by the age of two years of life [9]. Furthermore, RV can cause illnesses ranging from mild upper respiratory tract infections to more severe conditions such as bronchiolitis, asthma exacerbations, laryngeal croup, and community-acquired pneumonia, which may require hospitalization and, in some cases, mechanical ventilation support [10,11,12,13]. Although RV is usually known as the etiological agent of the common cold, it has been observed that it can also cause severe ARIs in children, older adults, and immunocompromised individuals [14] and has been associated with fatal cases [15,16,17].

RSV is an enveloped RNA virus with a non-segmented, negative-strand genome (ssRNA [−]) [18]. This virus belongs to the family *Pneumoviridae*, genus *Orthopneumovirus*, and the species *Orthopneumovirus hominis* [19]. Its genome is around 15.2K nucleotides and has 10 genes that encode 11 proteins [20]. Based on the differences in the region of attachment glycoprotein (G), RSV is classified into two major subgroups: RSV-A and RSV-B [21,22]. More recently, a consensus nomenclature system was proposed to standardize the reclassification of RSV genotypes into phylogenetic lineages within each subgroup [22]. Under this classification, RSV-A includes the lineages A.1, A.2, A.2.1, A.2.1.1, A.3, A.3.1, A.3.1.1, A.D, A.D.1, A.D.1.1–A.D.1.11, A.D.2, A.D.2.1, A.D.2.2, A.D.2.2.1, A.D.3, A.D.3.1–A.D.3.12, A.D.4, A.D.4.1, A.D.5, A.D.5.1–A.D.5.4, and RSV-B includes the lineages B, B.1–B.4, B.D, B.D.1, B.D.1.1, B.D.2, B.D.3, B.D.4, B.D.4.1, B.D.4.1.1–B.D.4.1.3, B.D.E.1, B.D.E.1.1–B.D.E.1.8, B.D.E.2–B.D.E.7 [22]. The current RSV nomenclature incorporates the molecular characteristics previously associated with the ON1 and BA genotypes, the 72 nucleotide duplication in RSV-A and the 60 nucleotide duplication in RSV-B within the G gene. These variants are now classified within the A.D and B.D lineages, respectively [22].

RV is a positive-sense single-stranded RNA (ssRNA [+]) virus with a genome of approximately 7.2K nt [23]. This virus belongs to the *Picornaviridae* family, *Enterovirus* genus, and is classified into three species: *Enterovirus alpharhino*, *Enterovirus betarhino*, and *Enterovirus cerhino*, formerly known as RV-A, RV-B, and RV-C, respectively [19]. Its genome comprises a single open reading frame (ORF), which is translated into a single polyprotein that is subsequently cleaved by viral proteases into 11 viral proteins [23]. These include four structural proteins (VP1, VP2, VP3, and VP4) and seven non-structural proteins: 2A (protease), 2B, 2C, 3A, 3B (viral initiation protein [VPg]), 3C (protease), and 3D (RdRp polymerase) [24]. To date, 169 RV genotypes have been identified and distributed among the three species, including 80 RV-A, 32 RV-B, and 57 RV-C genotypes [25]. This classification is mainly based on sequence analysis of the VP1-VP4 genomic regions [26].

With the emergence of severe acute respiratory syndrome coronavirus 2 (SARS-CoV-2) in late 2019 [27] and the subsequent declaration of the COVID-19 pandemic, governments across the globe implemented non-pharmaceutical interventions (NPIs), including lockdowns, mask use, social distancing, and mobility restrictions [28,29,30,31]. These interventions not only impacted the reduction or limitations of COVID-19 transmission but also altered the circulation patterns of other respiratory viruses, such as RSV and RV [31,32]. Numerous studies reported a marked reduction or near disappearance of RSV circulation during the early pandemic period, while RV continued to circulate at varying levels [31,32,33]. Following the relaxation of NPIs, several countries experienced atypical resurgences of RSV and changes in RV circulation, often outside their usual seasonal patterns [33,34,35,36].

Comparing the lineages/genotypes circulation of RSV and RV between the pandemic period (PP, 2020–2021) and the post-pandemic period (PPP, 2023–2024) provides an opportunity to assess how changes in human behavior shaped viral transmission. It also generates public health data to support the evaluation of interventions such as vaccination and other prevention and control measures.

In this study, we conducted a comparative molecular and epidemiological analysis of RSV and RV in Santander, Colombia, using respiratory samples collected during the PP and the PPP. By integrating RT-qPCR detection, phylogenetic analysis, and clinical data, we aimed to describe lineages/genotypes of RSV and RV in the region.

## 2. Materials and Methods

### 2.1. Study Population, Samples, and Data Collection

This study included respiratory samples collected from individuals presenting with ARI symptoms during two distinct periods: the pandemic period (PP, 2020–2021) and the post-pandemic period (PPP, 2023–2024). ARI was defined based on the presence of one or more symptoms at the time of sample collection, including fever, cough, fatigue, shortness of breath, sore throat, and loss of smell or taste, as recorded in clinical records.

During the PP, samples were collected between 9 April 2020 and 30 November 2021 from various municipalities throughout the department of Santander, Colombia. These specimens were initially gathered for SARS-CoV-2 (COVID-19) diagnostic testing and later stored at −80 °C in the Central Research Laboratory of the School of Microbiology at the Universidad Industrial de Santander. Residual aliquots from these stored samples were used for this study.

For the PPP, respiratory samples were collected between 2 March 2023 and 22 May 2024 from four healthcare facilities in Bucaramanga: Hospital Universitario de Santander (HUS), San Luis Clinic, Bucaramanga Health Institute (ISABU), Rosario Health Center, and ISABU Girardot Health Center, as well as from the Faculty of Health at Universidad Industrial de Santander.

Inclusion criteria for the PPP samples were individuals older than one year presenting with ARI symptoms of less than 15 days’ duration who either sought medical attention or were part of the university community. The exclusion of infants younger than one year was defined by the design of the broader study in which this work was nested, which aimed to compare saliva samples and nasopharyngeal swabs for the diagnosis of respiratory viral infections; saliva sample collection was not feasible in this age group. For the PP samples, both SARS-CoV-2-positive and –negative individuals with ARI symptoms were included if a stored aliquot of their respiratory sample was available. Additionally, clinical and epidemiological information was obtained by reviewing medical records for the sampled individuals.

### 2.2. Detection of RSV and RV

Nucleic acid extraction and purification were performed using the Quick-DNA/RNA Viral MagBead Kit (Zymo Research Corporation, Irvine, CA, USA) according to the manufacturer’s instructions.

For RSV detection, an approximately 96 bp fragment of the polymerase (L) gene was amplified using the following primers: forward 5′-AATACAGCCAAATCTAACCAACTTTACA-3′, reverse 5′-GCCAAGGAAGCATGCAATAAA-3′, and probes 5′-6-FAM-TGCTATTGT/ZEN/GCACTAAAG-3′-IABkFQ (RSV-A) and 5′-6-FAM-CACTATTCC/ZEN/TTACTAAAGATGTC-3′-IABkFQ (RSV-B) [37].

For RV detection, a ~114 bp fragment corresponding to the 5′ untranslated region (5′-UTR) of the viral genome was amplified using the primers described by Ng et al. (2016) [38]: forward 5′-GGCCCCTGAATGYGGCTAA-3′ and reverse 5′-GAAACACGGACACCCAAAGTAG-3′, together with a custom probe designed for this study: 5′-6-FAM-TCGTAAYGA/ZEN/GYAATTGCGGGAYGGRAC-3′-IABkFQ.

RT-qPCR assays were performed in a final reaction volume of 10 µL using the Luna^®^ Universal Probe One-Step RT-qPCR Kit (New England Biolabs, Ipswich, MA, USA). Each reaction contained 2 µL of RNA template, 5 µL of Luna Universal Probe One-Step Reaction Mix (2×), 0.16 µL of MgSO_4_ (50 mM; Invitrogen, Thermo Fisher Scientific, Waltham, MA, USA), 0.4 µL of each primer (10 µM), 0.2 µL of probe (10 µM), 0.5 µL of Luna WarmStart^®^ RT Enzyme Mix (20×) (New England Biolabs, Ipswich, MA, USA), and 1.34 µL of nuclease-free water. PCR amplifications for RSV, RV, and human RNase P (endogenous control) were conducted on a StepOnePlus™ Real-Time PCR System (Applied Biosystems, Foster City, CA, USA) under the following cycling conditions: reverse transcription at 55 °C for 10 min, initial denaturation at 95 °C for 1 min, followed by 40 cycles of 95 °C for 10 s and 60 °C for 1 min.

### 2.3. RT-PCR Amplification and Sequencing of the RSV Glycoprotein G Gene, and the RV VP4–VP2 Region

RSV- and RV-positive samples with a cycle threshold (Ct) value below 25 were selected for amplification of specific genomic regions for subsequent sequencing and phylogenetic analysis.

For RSV-positive samples, the near full-length gene (~966 bp for RSV-A and ~933 bp for RSV-B) encoding the attachment glycoprotein (G) was amplified using primers RSVA/B-G-F (5′-GGGCAAATGCAAACATGTCC-3′) and RSVA/B-G-R (5′-GCAACTCCATKGTTATTTGCC-3′) [39].

For RV-positive samples, the VP4–VP2 region was amplified using a nested PCR protocol. The first (outer) PCR was performed with primers fatal ari (5′-CCGGCCCCTGAATGYGGCTAA-3′) and OAS1125 (5′-ACATRTTYTSNCCAAANAYDCCCAT-3′), and the second (inner) PCR with primers IS547 (5′-ACCRACTACTTTGGGTGTCCGTG-3′) and IAS1087 (5′-TCWGGHARYTTCCAMCACCANCC-3′), generating an expected amplicon of approximately 544 bp [40].

RT-PCR reactions for RSV were carried out in a final volume of 25 µL using the LunaScript^®^ Multiplex One-Step RT-PCR Kit (New England Biolabs, Ipswich, MA, USA). Each reaction contained 5 µL of RNA template, 5 µL of 5× Reaction Mix, 11.5 µL of nuclease-free water, 1 µL of 25× Enzyme Mix, and 1.25 µL of each primer (10 µM). Amplifications were performed on a Veriti™ 96-Well Fast Thermal Cycler (Applied Biosystems, Foster City, CA, USA) under the following cycling conditions: reverse transcription at 55 °C for 10 min; initial denaturation at 98 °C for 1 min; 40 cycles of 98 °C for 10 s, 63 °C for 1 min, and 72 °C for 1 min; and a final extension at 72 °C for 5 min. PCR products were visualized by electrophoresis on a 1% agarose gel in 1× TAE buffer at 80 V for 1 h.

Nested RT-PCR amplification of the RV VP4–VP2 region was performed as follows. The first round used the same LunaScript^®^ Multiplex One-Step RT-PCR Kit (New England Biolabs, Ipswich, MA, USA) in 25 µL reactions containing 10 µL of RNA, 5 µL of 5× Reaction Mix, 5.8 µL of water, 1 µL of 25× Enzyme Mix, and 1.6 µL of each primer (10 µM). Cycling conditions were reverse transcription at 55 °C for 10 min; initial denaturation and reverse transcriptase inactivation at 98 °C for 1 min; 40 cycles of 98 °C for 10 s, 60.5 °C for 30 s, and 72 °C for 1 min; and a final extension at 72 °C for 5 min. The second PCR round was performed in 25 µL reactions containing 5 µL of the first-round product, 5 µL of 5× Reaction Mix, 10.8 µL of water, 1 µL of 25× Enzyme Mix, and 1.6 µL of each primer (10 µM). The cycling program was initial denaturation at 98 °C for 1 min; 35 cycles of 98 °C for 10 s, 65.5 °C for 30 s, and 72 °C for 30 s; and final extension at 72 °C for 5 min. PCR products were visualized on 2% agarose gels in 1× TAE buffer at 90 V for 80 min.

All RSV and RV amplicons were purified using the DNA Clean & Concentrator^®^-25 Kit (Zymo Research Corporation, Irvine, CA, USA) and sequenced bidirectionally with the same primers used for amplification (RSV: RSVA/B-G-F and RSVA/B-G-R; RV: IS547 and IAS1087). Sanger sequencing was performed on a SeqStudio™ Genetic Analyzer (Thermo Fisher Scientific, Waltham, MA, USA) by GENCELL PHARMA (Bogotá, Colombia). All RSV and RV sequences generated in this study have been deposited in GenBank under accession numbers PZ035431-PZ035440 (RSV) and PZ096310-PZ096327 (RV).

### 2.4. Phylogenetic Analysis of RSV and RV

To classify viral sequences at a lower level within the subgroup (lineage, in the case of RSV) and at the genotype level (in the case of RSV), reference sequences representative of each RSV-A and RSV-B lineage, as well as each RV-A, RV-B, and RV-C genotype, were retrieved from GenBank. Three independent datasets were constructed: one for RSV-A, one for RSV-B, and another for RV-A/B/C genotypes. A total of 277 RSV-A attachment glycoprotein (G) gene sequences representing 50 distinct lineages and 102 RSV-B sequences representing 24 lineages were included in the phylogenetic analysis; of these sequences, 135 RSV-A sequences corresponded to reference sequences reported by the RSV Genotyping Consensus Consortium (RGCC) and 102 RSV-B reference sequences [22]. Likewise, partial sequences of the glycoprotein G (G) gene previously reported from Colombia were included [41]. The accession numbers and metadata associated with these reference sequences obtained from GenBank are provided in Supplementary Table S1.

For RV, reference sequences representing 77 genotypes of RV-A (*n* = 151), 30 genotypes of RV-B (*n* = 51), and 45 genotypes of RV-C (*n* = 67) were obtained (Table S2). RV genotypes were classified based on the VP4–VP2 region using GenBank reference sequences with previously established assignments.

Multiple sequence alignments were generated with MAFFT v7 [42], using the most suitable strategy (L-INS-i, FFT-NS-i, or FFT-NS-2) based on dataset size. Alignments were manually refined in AliView v1.28 [43], the ends of each sequence were trimmed to remove low-quality regions and primer binding sites, retaining only the genomic regions of interest, and then realigned with MAFFT using default settings. The best-fit nucleotide substitution model for each dataset was determined with ModelFinder [44], implemented in IQ-TREE v3.1.2 [45]. Maximum-likelihood (ML) phylogenies were constructed with IQ-TREE, using 10,000 replicates of the Ultrafast Bootstrap approximation (UFBoot) [46], along with nearest-neighbor interchange (NNI) searches. Node support was further evaluated with the SH-like approximate likelihood ratio test (SH-aLRT) [47]. ML trees were visualized and annotated with FigTree v1.4.4 [48], and the ggtree package v3.12.0 in R [49,50,51,52,53].

### 2.5. Analysis of RSV and RV Amino Acid Sequences

Amino acid sequences of the RSV attachment glycoprotein (G) and the RV VP4–VP2 proteins were deduced using the Sequence Manipulation Suite translation tool (https://www.bioinformatics.org/sms2/translate.html, accessed on 25 May 2026). For RSV-A, seven sequences were used as a reference for each lineage: OQ171906.1 (A.D.1.5), OQ171911.1 (A.D.1.7), PP973760.1 (A.D.1.8), PP508181.1 (A.D.3.2), PP530269.1 (A.D.3.3), MZ516012.1 (A.D.5.1) and OK500260.1 (A.D.5.2). For RSV-B, one sequence was used as a reference for the BDE1 lineage (OP965703.1). For RV-A, RV-B, and RV-C, reference sequences NC_001617.1, NC_001490.1, and NC_009996.1, respectively, were incorporated.

Multiple sequence alignments were performed in MAFFT v7 [42] using default parameters. Amino acid substitutions were identified, analyzed, and visualized with Unipro UGENE v51.0 [54].

### 2.6. Statistical Analysis

A descriptive analysis was conducted for variables such as sex at birth, age, case type, symptoms, and comorbidities. Variables were summarized using absolute and relative frequencies. Comparisons between categorical variables were performed with the Chi-square test or Fisher’s exact test, as appropriate. Statistical significance was established at *p* < 0.05. All statistical analyses and figure creation were carried out in the software R using RStudio Version 4.3.2 (Posit team).

## 3. Results

### 3.1. Detection of RSV, RV, and Epidemiological and Clinical Characteristics of the Study Population

A total of 921 respiratory samples from patients with ARI symptoms were analyzed, including samples collected during the PP (between April 2020 and November 2021; *n* = 600) and the PPP (March 2023–May 2024; *n* = 321). Overall, RSV-A was detected in 2.0% of samples (18/921; 95%CI: 1.2–3.1), RSV-B in 1.5% (14/921; 95%CI: 0.9–2.6), and RV in 12.9% (119/921; 95% CI: 10.8–15.3).

**Figure 1:**
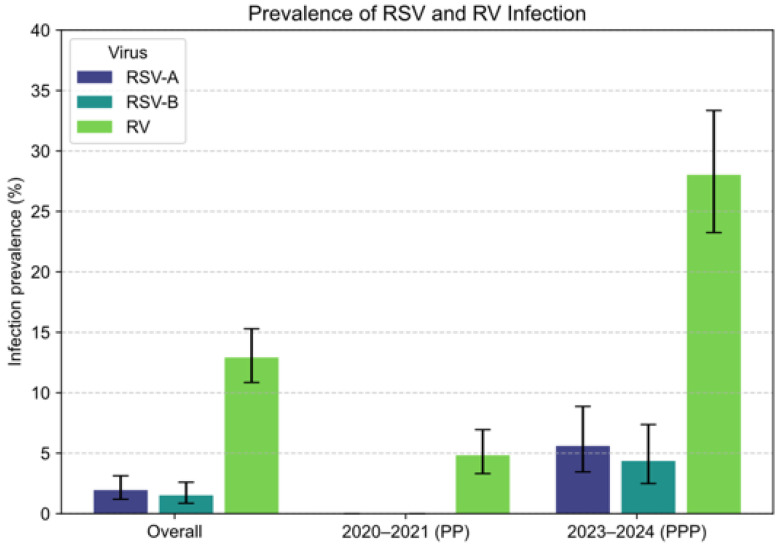
Prevalence of RSV-A, RSV-B, and RV infections during the pandemic and post-pandemic periods in Santander, Colombia. Prevalence (%) of RSV-A, RSV-B, and RV among patients with ARI in Santander, Colombia. A total of 921 respiratory samples were analyzed, including samples from the PP (April 2020–November 2021; *n* = 600) and the PPP (March 2023–May 2024; *n* = 321). Error bars represent 95% confidence intervals. RSV-A and RSV-B were not detected during the PP but were identified during the PPP, whereas RV was detected in both periods.

During the PP, no RSV-A or RSV-B infections were detected, whereas RV was identified in 4.8% of samples (*n* = 29/600; 95%CI: 3.3–7.0). In contrast, during the PPP, RSV-A was detected in 5.6% of samples (*n* = 18/321; 95%CI: 3.4–8.9), RSV-B in 4.4% (*n* = 14/321; 95%CI: 2.5–7.4), and RV in 28.0% (*n* = 90/321; 95%CI: 23.2–33.4) (Figure 1). Overall, RV prevalence was higher than RSV prevalence in both study periods. RSV detection was limited to samples collected during the PPP, whereas RV was detected during both periods.

The study population comprised 52.6% of male and 47.4% female patients, with ages ranging from 0 to 104 years (mean = 41.0 years; IQR = 42.0). The most represented age group was 25–64 years (45.7%), followed by individuals ≥ 65 years (23.6%). Most cases were managed on an outpatient basis (51.0%), while 36.3% required hospitalization, 5.9% were admitted to the intensive care unit (ICU), and 6.5% were fatal cases.

Among RSV-A positive cases, the most affected age group was 1–4 years (44.4%), and a statistically significant association between age group and infection was observed (*p* < 0.05). RSV-B infections were most frequent in children aged 5–14 years (35.7%) and 1–4 years (28.6%), also showing a significant association with age (*p* < 0.05). RV infections were distributed across all age groups and showed statistically significant associations with sex, age, case type, symptoms, and comorbidities (*p* < 0.05). Detailed epidemiological and clinical characteristics are presented in Table 1.

### 3.2. Phylogenetic Analysis of RSV

Through RT-qPCR analysis, 18 samples tested positive for RSV-A and 14 for RSV-B during the PPP. Of these, near full-length sequencing of the attachment glycoprotein G gene was successfully performed on 8 RSV-A and 2 RSV-B samples. Using sequences obtained, phylogenetic analysis was performed to determine the distribution of RSV-A and RSV-B lineages circulating during 2023–2024.

**Figure 2:**
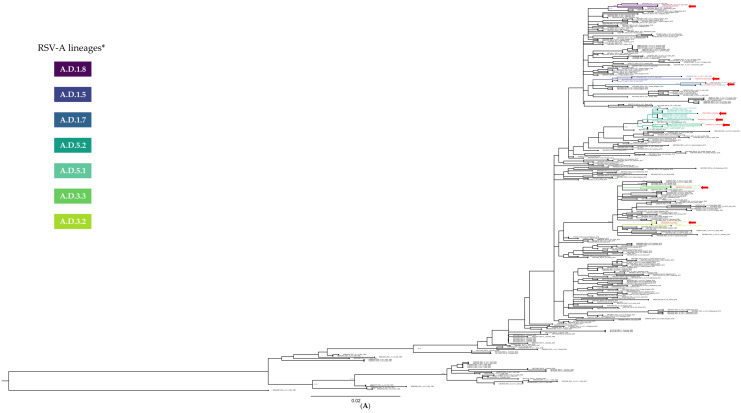

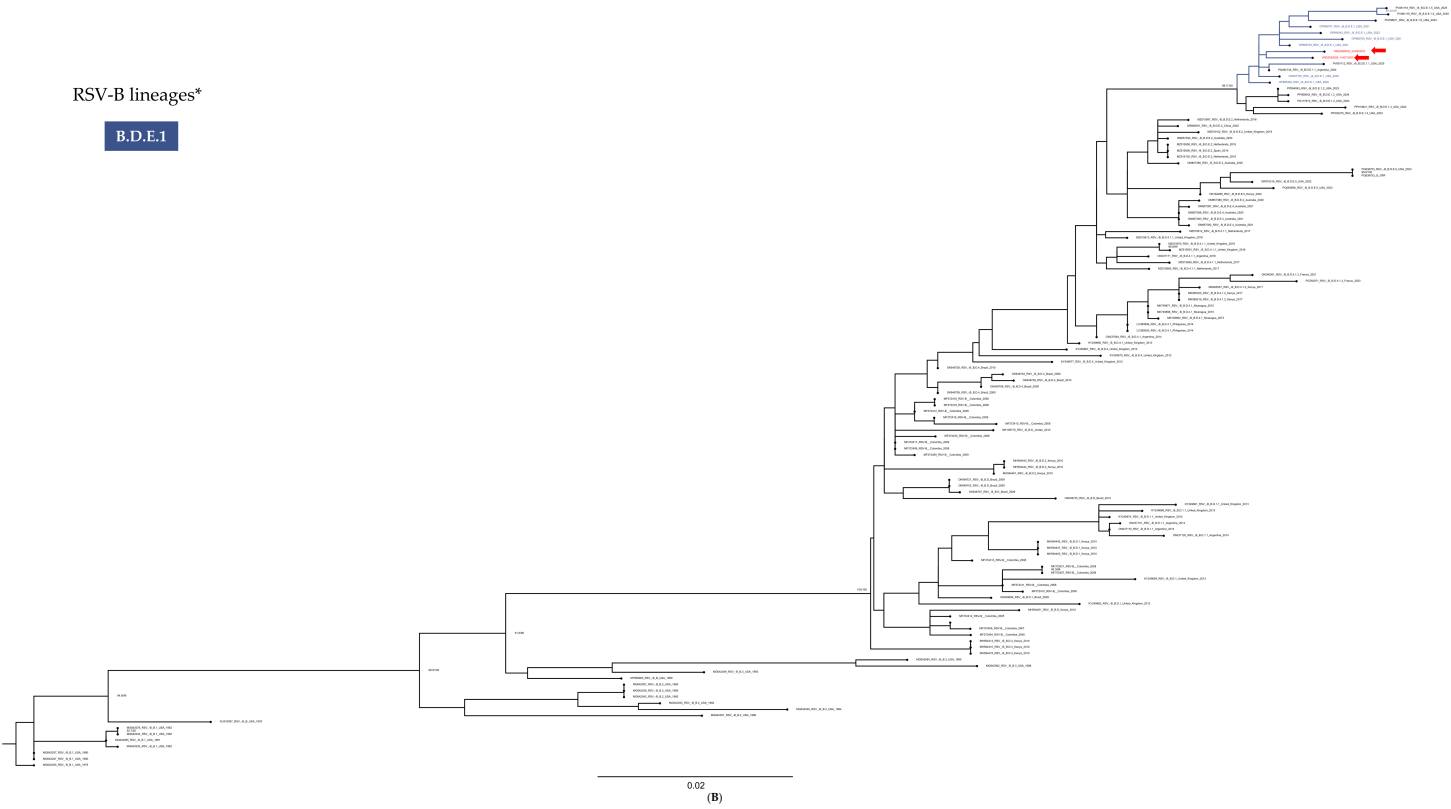
ML phylogenetic analysis of the RSV G gene. (**A**) RSV-A sequences and (**B**) RSV-B sequences obtained during the PPP period and reference sequences. Trees were reconstructed using the ML method with representative reference sequences of known lineages. Sequences generated in this study are indicated with red arrows. All RSV-A sequences were distributed among eight different lineages: A.D.1.5, A.D.1.7, A.D.1.8, A.D.3.2, A.D.3.3, A.D.5.1 and A.D.5.2, whereas RSV-B sequences grouped within the B.D.E.1 lineage. Bootstrap support values are shown at key nodes. The scale bar represents nucleotide substitutions per site. * lineages in which our sequences were clustered.

All RSV-A sequences from this study clustered within the A.D lineage (Figure 2A). These sequences were distributed across seven A.D-derived lineages: A.D.1.5 (*n* = 1), A.D.1.7 (*n* = 1), A.D.1.8 (*n* = 1), A.D.3.2 (*n* = 1), A.D.3.3 (*n* = 1), A.D.5.1 (*n* = 1), and A.D.5.2 (*n* = 2). Sequences within the A.D.1 group corresponded to the A.D.1.5, A.D.1.7, and A.D.1.8 lineages, whereas those within the A.D.3 group were assigned to A.D.3.2 and A.D.3.3. Within the A.D.5 group, one sequence was classified as A.D.5.1 and two as A.D.5.2. The RSV-A reference sequences included in the tree comprised isolates collected both before 2020 and after 2020. The RSV-A sequences from this study, collected between 2023 and 2024, clustered mainly with post-2020 references in the A.D.1.5, A.D.1.7, A.D.1.8, A.D.3.2, A.D.3.3, and A.D.5.2 lineages. In contrast, the sequence assigned to A.D.5.1 clustered with pre-pandemic references from 2019.

For RSV-B, the two sequences obtained in this study clustered within the B.D.E.1 lineage (Figure 2B). Both sequences formed a clade within this lineage and were near to post-2020 sequences from the United States collected between 2021 and 2022.

Additionally, besides the sequences reported by the RGCC, those already assigned to specific lineages, a group of previously reported RSV-A and RSV-B sequences from Colombia was included in the phylogenetic analysis. These Colombian reference sequences clustered within older lineages compared with the sequences obtained in this study. For RSV-A, previous Colombian sequences grouped within A.2.1, A.2.1.1, and A.3.1, whereas all RSV-A sequences from this study were assigned to A.D-derived lineages. For RSV-B, previous Colombian sequences clustered within B.D, B.D.1, and B.D.4, while the RSV-B sequences from this study grouped within B.D.E.1.

### 3.3. Amino Acidic Sequence Analysis of RSV

The amino acid sequence analysis of the attachment glycoprotein (G) was performed using one (or several) representative reference sequence(s) for each lineage identified in the RSV-A/B samples of this study. In the RSV-A sequences, specific amino acid substitutions were observed when compared with the corresponding reference sequence for each lineage. The sequence VRES020199, classified as A.D.1.5, presented the changes H67N, L71V, Y102S, T264I, T282P, G296E, and S313Y. In the A.D.1.7 lineage, the sequence VRES020066 presented the changes Q120P, K192R, and K271E, whereas the sequence VRES020129, classified as A.D.1.8, presented the changes I114M, A137T, R209K, A225V, K287E, T319K, and T320A. In the A.D.3.2 lineage, the sequence VRES020094 showed the changes S177N, I230T, and P247L. In the case of lineages with previously reported changes in the attachment glycoprotein (G) by Goya et al. [22], the sequence VRES020150, classified as A.D.3.3, presented the N204 change, coinciding with the marker reported for this lineage. In addition, this sequence showed the substitutions L97F, P111S, A122D, T200I, S234P, and K251N with respect to the reference used. Similarly, the sequence VRES020131, classified as A.D.5.1, presented the I118 change, consistent with the change reported for this lineage, together with the additional substitutions Q127L, P276L, E295K, and S313Y. The two sequences classified as A.D.5.2 presented different amino acid profiles. VRES020100 presented A47G, I59V, L71P, and S105F, whereas VRES340008 presented L24V, R151H, T219N, T235I, S277P, S294P, G296S, Y297H, and P300L. Although both sequences belonged to the same lineage, they did not share the same substitution pattern with respect to the reference sequence used.

For the amino acid analysis of RSV-B, a representative sequence of the B.D.E.1 lineage was used as reference: OP965703.1. When comparing the two RSV-B sequences from this study with the OP965703.1 reference, punctual amino acid substitutions were observed within the attachment glycoprotein (G). The sequence VRES020058 presented the substitutions S115P, S255P, L265S, and T300I, whereas VRES060002 presented only the L265S substitution. Among the B.D.E.1 reference sequence included in the alignment, variations not previously described as lineage-specific markers were also observed. In particular, the L265S change was present in all B.D.E.1 reference sequences compared with OP965703.1, as well as in both sequences from this study. In addition, other changes were observed, including Q104R, R98K, T139I, P152S, I173V, and V249A.

Additionally, amino acid variations were observed in some reference sequences included in the alignment that do not correspond to the punctual changes previously reported as lineage markers. A summary of the reference sequences used, the changes reported for each lineage, and the substitutions observed in these study sequences is presented in Table 2.

### 3.4. Phylogenetic and Amino Acid Analysis of RV

**Figure 3:**
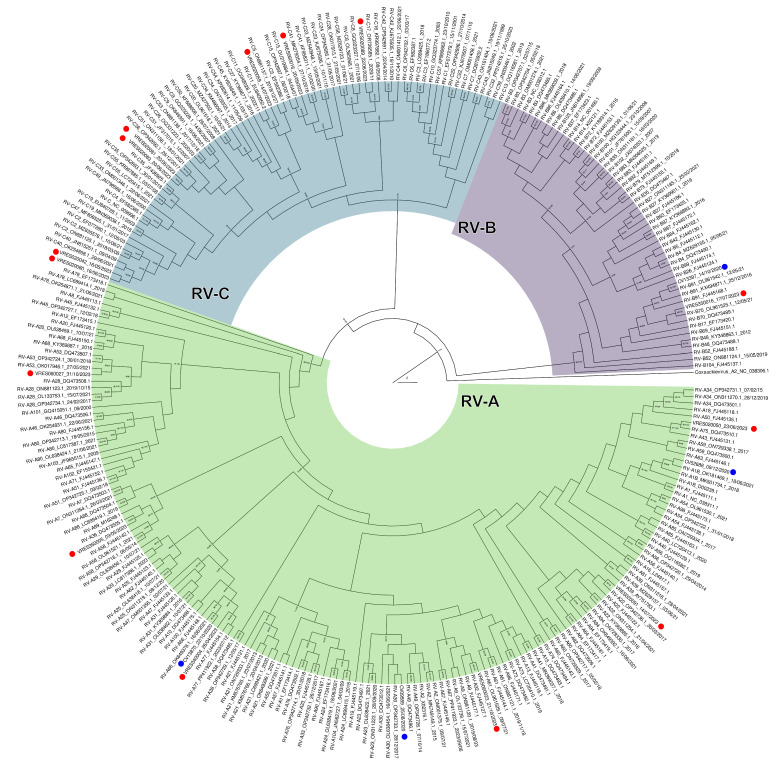
Maximum likelihood phylogenetic tree inferred from rhinovirus (RV) VP4–VP2 region sequences. The tree shows the genetic relationships between RV-A, RV-B, and RV-C species, which are indicated by shaded sectors. Sequences generated in this study are marked with circles: blue for PP sequences and red for PPP sequences. Sequences generated in this study are marked with red circles. During the PP, both RV-A and RV-B species were detected, corresponding to genotypes RV-A30, RV-A66, RV-A1B, and RV-B91. In contrast, greater genotype diversity was observed during the PPP, with identification of six RV-A genotypes (A22, A77, A75, A53, A36, and A32), one RV-B genotype (B70), and five RV-C genotypes (C11, C15, C17, C40, and C46). The tree was reconstructed using the maximum likelihood method, and branch lengths represent nucleotide substitutions per site.

During the PP, RV was detected in 4.8% (*n* = 29) of the analyzed samples, and of these, 13.8% (*n* = 4) were successfully sequenced. ML phylogenetic analysis revealed that both RV-A and RV-B species circulated during this time, specifically genotypes RV-A30, RV-A66, RV-A1B, and RV-B91 (Figure 3). In contrast to the 2020–2021 period, greater genotype diversity was observed during the PPP. During this time, RV was detected in 28.0% of the analyzed samples, and the VP4–VP2 region was successfully sequenced in 15.5% of cases. A total of 12 distinct RV genotypes were identified, including six RV-A genotypes (A22, A77, A75, A53, A36, and A32); one RV-B genotype (B70); and five RV-C genotypes (C11, C15, C17, C40 and C46).

**Figure 4:**
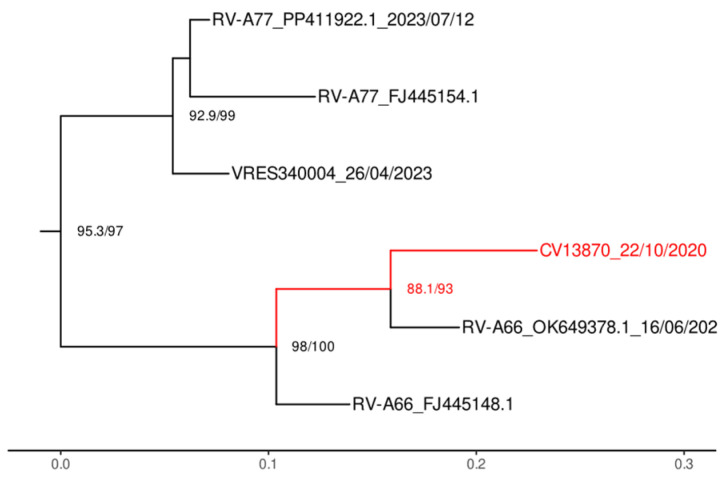
Maximum likelihood phylogenetic tree based on the rhinovirus VP4–VP2 region, highlighting the fatal acute respiratory infection (ARI) case detected during the PP. The sequence generated in this study (CV13870_22/10/2020) is shown in red. Phylogenetic analysis demonstrated that the study sequence clustered within the RV-A66 genotype clade, grouping closely with reference sequences OK649378.1 and FJ445148.1. Numbers at the nodes indicate bootstrap support values, and branch lengths represent nucleotide substitutions per site.

One fatal ARI case associated with RVA66 infection was identified during the PP in a 14-year-old male patient with underlying comorbidities (Figure 4). Phylogenetic analysis showed that the sequence clustered closely with RV-A66 genotype sequences (OK649378.1 and FJ445148.1).

A detailed comparison of the sequences from this study with those reported from other countries and their collection dates showed that sequences collected during the PP were closely related to sequences from the United States, Japan, and China, reported in 2019 and 2020. Sequences from the PPP were related to those previously reported in countries such as the United States, China, and Thailand, primarily from the years 1959, 2006, 2007, 2009, 2019, and 2023. Analysis of the nucleotide and deduced amino acid sequences of the VP4–VP2 region revealed a characteristic feature in RV-C strains: a six-nucleotide deletion resulting in the loss of two amino acids in the protein, Gly64del and Ile/Ala65del.

## 4. Discussion

Following the emergence of SARS-CoV-2 in late 2019 and the declaration of the COVID-19 pandemic in 2020 [30], governments worldwide implemented non-pharmaceutical interventions (NPIs), including strict lockdowns, mask use, social distancing, and mobility restrictions, which profoundly altered the transmission dynamics of respiratory viruses [31]. Globally, a marked reduction or near disappearance of RSV circulation was reported during the early pandemic period, whereas RV continued to circulate at variable levels, and atypical resurgences of both viruses were observed after relaxation of NPIs [32]. In Colombia, although a proportion of ARI cases during 2020–2022 were attributable to viruses other than SARS-CoV-2, including RSV and RV [55], our study found no RSV detection in samples collected during the PP, while RV was detected in 4.8% of cases. In contrast, during the PPP, higher detection frequencies of RSV (10.0%) and RV (28.0%) were observed. Together, these findings support a differential impact of COVID-19 NPIs on the circulation of RSV and RV in this region. As observed in other countries, as biosafety measures were relaxed, many regions of the world experienced off-season resurgences of respiratory pathogens, especially RSV and RV [33,34,35,36].

Our data show that children older than one year and younger than five were especially susceptible to RSV infection. A statistically higher proportion of patients aged 1 to 4 years with RSV-A infection and 5 to 14 years with RSV-B infection was found compared to other age groups. Globally, it has been reported that, in the years following the onset of the COVID-19 pandemic, the proportion of patients aged two years or older who became infected with RSV increased [56,57]. This increase in cases, already reported in Taiwan, France, the United Kingdom, and Argentina [56,58,59,60,61], demonstrated that children were more susceptible due to the lack of exposure to the virus during the pandemic period, which reduced immunity. It was reported that antibody titers in 2021 samples were up to 15 times lower than those from 2020 [62]. This phenomenon, often referred to as “immune debt,” reflects decreased herd immunity following prolonged periods of low circulation and is associated with increased likelihood of larger or atypical epidemics [63].

In Colombia, RSV-A and RSV-B genotypes circulating before the COVID-19 pandemic have been previously described [41,64]. During 2000–2009, the formerly known GA2 and GA5 genotypes (RSV-A) and BA genotype (RSV-B) were the most prevalent [41], whereas ON1, now classified as the A.D lineage, and BA, now classified within B.D-derived lineages, were detected in 2016–2017 [64]. Reclassification of these Colombian sequences placed pre-ON1 RSV-A genotypes within non-A.D-derived lineages, including A.2.1, A.2.1.1, and A.3.1, while RSV-B sequences clustered within B.D, B.D.1, and B.D.4 lineages. In our dataset, RSV-A sequences belonged to A.D-derived lineages and RSV-B sequences to B.D-derived lineages, supporting the continued circulation of these globally successful lineages in Colombia. Since their initial descriptions—ON1 in Canada in 2010 and BA in Argentina in the 1990s—these genotypes have circulated globally without clear regional restriction [65,66]. Thus, our findings support that RSV diversity is mainly shaped by temporal lineage replacement rather than geographic structure [67] as reflected by the absence of formerly circulating lineages in our dataset and the clustering of our sequences with recently circulating global lineages [41,64].

Consistent with previous studies, most of the nucleotide variability in RSV was observed in the attachment glycoprotein (G) gene [68,69]. The A.D and B.D lineages, defined by 72- and 60-nucleotide duplications in the G gene, respectively, continue to diversify through accumulation of amino acid substitutions, particularly within the second hypervariable region [65,69,70]. In our dataset, the detection of multiple A.D and B.D derived lineages, together with the amino acid substitutions observed in the G protein alignment, reflects the genetic variability present among RSV sequences collected during PPP. This divergence, especially of A.D strains, has been associated with increased clinical severity of bronchiolitis [71] and is thought to be driven by immune pressure on the G glycoprotein, showing that the emergence of these new lineages favors variants capable of evading host immunity [70,72]. Similarly, RSV sequences analyzed in this study showed multiple SNPs resulting in amino acid substitutions in the G protein compared with reference sequences, particularly within the second hypervariable region, which is known to exhibit high variability. Nevertheless, it has been documented that an increasing number of amino acid substitutions in this protein region may contribute to immune escape and/or increased virulence [72].

RV has long been recognized as the primary causative agent of the common cold [24], but it is increasingly associated with severe ARIs, asthma exacerbations, and fatal outcomes [15]. In this study, RV continued to be detected during the PP, whereas an increase in detection frequency and genotype diversity was observed during PPP. The higher persistence of RV compared with RSV during the pandemic may be related, in part, to its non-enveloped structure, which confers greater environmental stability [73,74]. During PP, only RV-A and RV-B genotypes were detected, and no RV-C strains were identified, despite reports indicating that RV-A and RV-C are typically the most prevalent species [75]. Multiple studies have reported that RV was the main respiratory virus cocirculating with SARS-CoV-2 during the pandemic [2,76,77,78]. It has been proposed that RV infections in humans could have a mutualistic effect. From the virus’s perspective, the immune response induced by RV allows for its replication and transmission; however, this same antiviral response could also inhibit infection by other potentially more lethal viruses such as SARS-CoV-2, which may explain its persistence and high prevalence during a period when the circulation of other viruses was restricted [79]. Similar viral interference has been described for RV and influenza A virus during the 2009 H1N1 pandemic, where RV circulation was proposed to have contributed to disruption of influenza spread in Europe [80].

During the PPP, RV detection increased, and genotypes from all three species (RV-A, RV-B, RV-C) were identified, with an overall higher number of genotypes than during the PP. Compared with the four genotypes detected in 2020–2021, twice as many genotypes were identified in 2023–2024, and none overlapped between periods. Consistent with previous reports, RV-A and RV-C were the most frequently detected species after the relaxation of NPIs, likely reflecting changes in transmission opportunities during the PPP [81,82].

Phylogenetic analysis further showed that RV diversity was influenced by temporal distribution, as sequences from the PP were most closely related to lineages reported in previous years, whereas post-pandemic sequences clustered with contemporaneous global strains. However, no dominant outbreak genotype was identified.

RV is an RNA virus with a high mutation rate [83]. The analysis of nucleotide sequences revealed that RV variation was mainly due to SNP-type variation events. These changes in protein-coding regions exposed to the immune system and involved in cell entry (such as VP2 and VP4) may promote immune evasion, allowing various genotypes to persist over time and diversify into different clades [26].

Overall, this study highlights the importance of molecular surveillance to understand the circulation of endemic respiratory viruses. To our knowledge, this is the first study in Colombia to compare RSV and RV genetic diversity before and after the COVID-19 pandemic and the first to characterize RV genotypes in the country. These findings provide a framework for strengthening virological surveillance and informing preparedness and prevention strategies in post-pandemic settings. This study has several limitations. First, samples were obtained through convenience surveillance, which may not fully represent community-wide circulation patterns in the region. Second, the number of RSV- and RV-positive samples successfully sequenced was low, which may reduce the resolution of phylogenetic inferences. Third, only partial genomic regions were analyzed, specifically the RSV G gene and the RV VP4–VP2 region, whereas whole-genome sequencing would provide a more comprehensive view of viral evolution. Despite the limitations of this study, it provides relevant epidemiological and molecular information on RSV and RV circulation in Santander, Colombia.

## 5. Conclusions

The COVID-19 pandemic and the implementation of non-pharmaceutical interventions altered the circulation of respiratory viruses in this region. RSV was not detected in PP but re-emerged with higher detection frequency in the PPP, particularly among children, whereas RV continued to circulate throughout the pandemic. Phylogenetic analysis showed that RSV-A sequences clustered within the A.D.1.5, A.D.1.7, A.D.1.8, A.D.3.2, A.D.3.3, A.D.5.1, and A.D.5.2 lineages, while RSV-B sequences clustered within the B.D.E.1 lineage. For RV, only RV-A and RV-B genotypes were detected during the PP, whereas genotypes from all three species (RV-A, RV-B, and RV-C) were identified after relaxation of NPIs during the PPP.

Although most cases were managed on an outpatient basis, a subset required hospitalization or ICU admission, and one fatal case associated with RV-A66 in a child with asthma underscored the potential severity of RV infection.

To our knowledge, this is the first study in Colombia to compare the genetic diversity of RSV and RV during and after the COVID-19 pandemic. These findings highlight the value of sustained molecular surveillance to monitor long-term epidemiological and evolutionary impacts of large-scale public health interventions and to inform future prevention and control strategies.

## Figures and Tables

**Table 1:** Demographic and clinical characteristics of the study population according to RV and RSV infection status.

Categories		Study Population						
		Total Population	RV-Positive Samples		RSV-Positive Samples			
					RSV-A		RSV-B	
		% (*n*)	% (*n*)	*p* Value	% (*n*)	*p* Value	% (*n*)	*p* Value
Sex	Male	52.6 (484)	40.3 (48)	0.004	44.4 (8)	0.487	57.1 (8)	0.729
	Female	47.4 (437)	59.7 (71)		55.6 (10)		42.8 (6)	
Age	<1 year	0.3 (3)	0.0 (0)	<0.001	0.0 (0)	<0.001	0.0 (0)	<0.001
	1–4 years	6.2 (57)	16.8 (20)		44.4 (8)		28.6 (4)	
	5–14 years	13.8 (127)	26.9 (32)		38.9 (7)		35.7 (5)	
	15–24 years	10.4 (96)	22.7 (27)		0.0 (0)		7.1 (1)	
	25–64 years	45.7 (421)	29.4 (35)		11.1 (2)		28.6 (4)	
	>65 years	23.6 (217)	4.2 (5)		5.6 (1)		0.0 (0)	
Case type	Outpatient	51.0 (470)	75.6 (90)	<0.001	83.3 (15)	0.132	71.4 (10)	0.434
	Hospitalization	36.3 (334)	20.2 (24)		16.7 (3)		21.4 (3)	
	Fatal case	6.5 (60)	1.7 (2)		0.0 (0)		0.0 (0)	
	ICU	5.9 (54)	1.7 (2)		0.0 (0)		7.1 (1)	
	Missing data	0.3 (3)	0.8 (1)		0.0 (0)		0.0 (0)	
Symptoms	Cough	78.8 (726)	85.7 (102)	<0.001	100.0 (18)	0.070	92.8 (13)	0.657
	Fever	59.7 (550)	56.3 (67)		72.2 (13)		71.4 (10)	
	Dyspnea	44.2 (407)	37.8 (45)		33.3 (6)		64.3 (9)	
	Fatigue	39.2 (361)	34.4 (41)		11.1 (2)		14.3 (2)	
	Sore throat	38.3 (353)	46.2 (55)		33.3 (6)		50.0 (7)	
	Headache	36.5 (336)	48.7 (58)		33.3 (6)		42.8 (6)	
	Rhinorrhea	20.1 (185)	40.3 (48)		50.0 (9)		28.6 (4)	
	Malaise	17.6 (162)	17.6 (21)		5.6 (1)		0.0 (0)	
	Gastrointestinal symptoms	12.9 (119)	11.8 (14)		11.1 (2)		21.4 (3)	
	Myalgia	4.7 (43)	2.5 (3)		0.0 (0)		0.0 (0)	
	Ageusia	4.4 (41)	5.9 (7)		0.0 (0)		7.1 (1)	
	Anosmia	4.4 (41)	5.0 (6)		0.0 (0)		7.1 (1)	
	Other symptoms	14.4 (133)	8.4 (10)		0.0 (0)		7.1 (1)	
Comorbidities	No comorbidities	52.2 (480)	68.1 (81)	<0.001	83.3 (15)	0.163	71.4 (10)	0.061
	Cardiovascular disease	19.2 (177)	8.4 (10)		5.6 (1)		0.0 (0)	
	Diabetes	10.6 (98)	1.7 (2)		0.0 (0)		0.0 (0)	
	Obesity	8.0 (74)	5.0 (6)		0.0 (0)		0.0 (0)	
	COPD	6.1 (56)	1.7 (2)		0.0 (0)		0.0 (0)	
	Asthma	5.6 (52)	11.8 (14)		5.6 (1)		21.4 (3)	
	Smoking	4.9 (45)	2.5 (3)		0.0 (0)		0.0 (0)	
	Pregnancy	1.4 (13)	0.8 (1)		0.0 (0)		0.0 (0)	
	Cancer	1.4 (13)	0.0 (0)		0.0 (0)		0.0 (0)	
	Immunosuppression	0.5 (5)	0.8 (1)		0.0 (0)		0.0 (0)	
	Tuberculosis	0.4 (4)	0.0 (0)		0.0 (0)		0.0 (0)	
	HIV	0.2 (2)	0.0 (0)		0.0 (0)		0.0 (0)	
	Other comorbidities	19.2 (177)	11.8 (14)		11.1 (2)		7.1 (3)	
	Missing data	0.4 (4)	0.0 (0)		0.0 (0)		0.0 (0)	

Distribution of demographic variables (sex and age group), clinical characteristics (case type and reported symptoms), and comorbidities among the total study population (*n* = 921), RV-positive cases, and RSV-positive cases stratified by subtype (RSV-A and RSV-B). Values are presented as percentage and number of cases (% [*n*]). *p* values indicate comparisons between virus-positive and virus-negative individuals within each category. RV, rhinovirus; RSV, respiratory syncytial virus; RSV-A, respiratory syncytial virus subtype A; RSV-B, respiratory syncytial virus subtype B; ICU, intensive care unit; COPD, chronic obstructive pulmonary disease; HIV, human immunodeficiency virus.

**Table 2:** Amino acid changes observed in the RSV sequences from the study compared to a representative reference sequence for each lineage.

Virus Group	Lineage	Reference Sequence	This Study Sequence	Previously Reported Lineage-Associated Change in G (Reference)	Amino Acid Changes/States Observed in Study Sequence	Additional Amino Acid Changes Observed in Reference Sequences not Previously Reported as Lineage Markers
RSV-A	A.D.1.5	OQ171906.1	VRES020199	Not reported	H67N, L71V, Y102S, T264I, T282P, G296E, S313Y	Y102S, A57T, S100N, S294P, F101L, L104P, N153I, G254R
RSV-A	A.D.1.7	OQ171911.1	VRES020066	Not reported	Q120P, K192R, K271E	Q120P, K271E, S44Y, E123K, K145N, V224I, S260N
RSV-A	A.D.1.8	PP973760.1	VRES020129	Not reported	I114M, A137T, R209K, A225V, K287E, T319K, T320A	A137T, R209K, A225V, K287E, T320A, L265P, Y273H, P276S
RSV-A	A.D.3.2	PP508181.1	VRES020094	Not reported	S177N, I230T, P247L	I230T, P247L, L97F, L298X
RSV-A	A.D.3.3	PP530269.1	VRES020150	G204 = N	L97F, P111S, A122D, T200I, **N204**, S234P, K251N	Not detected among the additional reference sequences analyzed
RSV-A	A.D.5.1	MZ516012.1	VRES020131	G118 = I	**I118**, Q127L, P276L, E295K, S313Y	Q127L, P276L, T136I, Q253K
RSV-A	A.D.5.2	OK500260.1	VRES020100	Not reported	A47G, I59V, L71P, S105F	T235I, T306A, G272D, G232R
RSV-A	A.D.5.2	OK500260.1	VRES340008	Not reported	L24V, R151H, T219N, T235I, S277P, S294P, G296S, Y297H, P300L	T235I, T306A, G272D, G232R
RSV-B	B.D.E.1	OP965703.1	VRES020058	Not reported	S115P, S255P, L265S, T300I	L265S, Q104R, R98K, T139I, P152S, I173V, V249A
RSV-B	B.D.E.1	OP965703.1	VRES060002	Not reported	L265S	L265S, Q104R, R98K, T139I, P152S, I173V, V249A

Previously reported lineage-associated changes in the G protein are indicated when available. Amino acid changes shown in bold correspond to positions that matched previously reported lineage-associated markers. Additional amino acid changes observed among reference sequences are included when they were not previously reported as lineage-associated markers.

## Data Availability

All RSV and RV sequences generated in this study have been deposited in GenBank under accession numbers PZ035431–PZ035440 (RSV) and PZ096310–PZ096327 (RV).

## Supplementary Materials

The following supporting information can be downloaded at: https://www.mdpi.com/article/10.3390/v18060666/s1, Table S1: RSV-A and RSV-B sequences used in phylogenetic analysis; Table S2: RV-A, RV-B and RV-C sequences used in phylogenetic analysis. Table S3: RSV-A and RSV-B reference sequences with collection date and geographic origin used for temporal analyses. Table S4: RV-A, RV-B and RV-C reference sequences with collection date and geographic origin used for temporal analyses.
