# Supplementary material for: Molecular Epidemiology of Respiratory Syncytial Virus and Rhinovirus in Santander, Colombia, During the COVID-19 Pandemic and Post-Pandemic Periods, 2020–2024
Source: Viruses. 2026 Jun 12;18(6):666. doi: 10.3390/v18060666 (PMC13307640; doi:10.3390/v18060666)
Supplement: Supplementary file 1 [file viruses-18-00666-s001.zip › viruses-4222259-updated final SI.pdf]

Table S1. RSV-A and RSV-B sequences used in phylogenetic analysis. This table lists the reference sequences retrieved from GenBank and used for phylogenetic reconstruction of RSV-A and RSV-B strains. For each sequence is presented the viral genus, species, virus name, GenBank accession number and assigned genotype are provided. These sequences were selected to represent the genetic diversity of circulating RSV genotypes and to support clade and subclade classification in the present study.

Genus	Species	Virus Name	Accession Number	Genotype
Orthopneumovirus	*Orthopneumovirus hominis*	Respiratory syncytial virus A	AF065257.1	GA1
Orthopneumovirus	*Orthopneumovirus hominis*	Respiratory syncytial virus A	AF065407.1	GA1
Orthopneumovirus	*Orthopneumovirus hominis*	Respiratory syncytial virus A	AF233902.1	GA1
Orthopneumovirus	*Orthopneumovirus hominis*	Respiratory syncytial virus A	AF233914.1	GA1
Orthopneumovirus	*Orthopneumovirus hominis*	Respiratory syncytial virus A	AF233917.1	GA1
Orthopneumovirus	*Orthopneumovirus hominis*	Respiratory syncytial virus A	JF920069.1	GA1
Orthopneumovirus	*Orthopneumovirus hominis*	Respiratory syncytial virus A	M11486.1	GA1
Orthopneumovirus	*Orthopneumovirus hominis*	Respiratory syncytial virus A	Z33427.1	GA1
Orthopneumovirus	*Orthopneumovirus hominis*	Respiratory syncytial virus A	Z33431.1	GA1
Orthopneumovirus	*Orthopneumovirus hominis*	Respiratory syncytial virus A	Z33432.1	GA1
Orthopneumovirus	*Orthopneumovirus hominis*	Respiratory syncytial virus A	AF065256.1	GA2
Orthopneumovirus	*Orthopneumovirus hominis*	Respiratory syncytial virus A	AF233900.1	GA2
Orthopneumovirus	*Orthopneumovirus hominis*	Respiratory syncytial virus A	AF233915.1	GA2
Orthopneumovirus	*Orthopneumovirus hominis*	Respiratory syncytial virus A	AF233923.1	GA2
Orthopneumovirus	*Orthopneumovirus hominis*	Respiratory syncytial virus A	AF448498.1	GA2
Orthopneumovirus	*Orthopneumovirus hominis*	Respiratory syncytial virus A	AY114149.1	GA2
Orthopneumovirus	*Orthopneumovirus hominis*	Respiratory syncytial virus A	AY114151.1	GA2
Orthopneumovirus	*Orthopneumovirus hominis*	Respiratory syncytial virus A	AY146435.1	GA2
Orthopneumovirus	*Orthopneumovirus hominis*	Respiratory syncytial virus A	AY472086.1	GA2
Orthopneumovirus	*Orthopneumovirus hominis*	Respiratory syncytial virus A	Z33422.1	GA2
Orthopneumovirus	*Orthopneumovirus hominis*	Respiratory syncytial virus A	AF233905.1	GA3
Orthopneumovirus	*Orthopneumovirus hominis*	Respiratory syncytial virus A	AF233913.1	GA3
Orthopneumovirus	*Orthopneumovirus hominis*	Respiratory syncytial virus A	AF233920.1	GA3
Orthopneumovirus	*Orthopneumovirus hominis*	Respiratory syncytial virus A	AF233921.1	GA3
Orthopneumovirus	*Orthopneumovirus hominis*	Respiratory syncytial virus A	Z33414.1	GA3

Orthopneumovirus	*Orthopneumovirus hominis*	Respiratory syncytial virus A	Z33416.1	GA3
Orthopneumovirus	*Orthopneumovirus hominis*	Respiratory syncytial virus A	Z33426.1	GA3
Orthopneumovirus	*Orthopneumovirus hominis*	Respiratory syncytial virus A	AF065254.1	GA4
Orthopneumovirus	*Orthopneumovirus hominis*	Respiratory syncytial virus A	AB175815.1	GA5
Orthopneumovirus	*Orthopneumovirus hominis*	Respiratory syncytial virus A	AF065255.1	GA5
Orthopneumovirus	*Orthopneumovirus hominis*	Respiratory syncytial virus A	AF233903.1	GA5
Orthopneumovirus	*Orthopneumovirus hominis*	Respiratory syncytial virus A	AF233906.1	GA5
Orthopneumovirus	*Orthopneumovirus hominis*	Respiratory syncytial virus A	AF233909.1	GA5
Orthopneumovirus	*Orthopneumovirus hominis*	Respiratory syncytial virus A	AF233916.1	GA5
Orthopneumovirus	*Orthopneumovirus hominis*	Respiratory syncytial virus A	AF233919.1	GA5
Orthopneumovirus	*Orthopneumovirus hominis*	Respiratory syncytial virus A	AF348803.1	GA5
Orthopneumovirus	*Orthopneumovirus hominis*	Respiratory syncytial virus A	AY114150.1	GA5
Orthopneumovirus	*Orthopneumovirus hominis*	Respiratory syncytial virus A	AY146437.1	GA5
Orthopneumovirus	*Orthopneumovirus hominis*	Respiratory syncytial virus A	AY472094.1	GA5
Orthopneumovirus	*Orthopneumovirus hominis*	Respiratory syncytial virus A	Z33430.1	GA5
Orthopneumovirus	*Orthopneumovirus hominis*	Respiratory syncytial virus A	Z33494.1	GA5
Orthopneumovirus	*Orthopneumovirus hominis*	Respiratory syncytial virus A	AF233901.1	GA6
Orthopneumovirus	*Orthopneumovirus hominis*	Respiratory syncytial virus A	AF233918.1	GA6
Orthopneumovirus	*Orthopneumovirus hominis*	Respiratory syncytial virus A	AF233904.1	GA7
Orthopneumovirus	*Orthopneumovirus hominis*	Respiratory syncytial virus A	AF233907.1	GA7
Orthopneumovirus	*Orthopneumovirus hominis*	Respiratory syncytial virus A	AF233910.1	GA7
Orthopneumovirus	*Orthopneumovirus hominis*	Respiratory syncytial virus A	AF348804.1	GA7
Orthopneumovirus	*Orthopneumovirus hominis*	Respiratory syncytial virus A	Z33417.1	GA7
Orthopneumovirus	*Orthopneumovirus hominis*	Respiratory syncytial virus A	Z33455.1	GA7
Orthopneumovirus	*Orthopneumovirus hominis*	Respiratory syncytial virus A	AB470478.1	NA1
Orthopneumovirus	*Orthopneumovirus hominis*	Respiratory syncytial virus A	KF300972.1	NA1
Orthopneumovirus	*Orthopneumovirus hominis*	Respiratory syncytial virus A	KP792358.1	NA1
Orthopneumovirus	*Orthopneumovirus hominis*	Respiratory syncytial virus A	KP792359.1	NA1
Orthopneumovirus	*Orthopneumovirus hominis*	Respiratory syncytial virus A	AB470478.1	NA1
Orthopneumovirus	*Orthopneumovirus hominis*	Respiratory syncytial virus A	DQ289605.1	NA1
Orthopneumovirus	*Orthopneumovirus hominis*	Respiratory syncytial virus A	DQ289633.1	NA1

Orthopneumovirus	*Orthopneumovirus hominis*	Respiratory syncytial virus A	FJ210826.1	NA1
Orthopneumovirus	*Orthopneumovirus hominis*	Respiratory syncytial virus A	FJ210829.1	NA1
Orthopneumovirus	*Orthopneumovirus hominis*	Respiratory syncytial virus A	FJ210830.1	NA1
Orthopneumovirus	*Orthopneumovirus hominis*	Respiratory syncytial virus A	GU550471.1	NA1
Orthopneumovirus	*Orthopneumovirus hominis*	Respiratory syncytial virus A	KJ710390.1	NA1
Orthopneumovirus	*Orthopneumovirus hominis*	Respiratory syncytial virus A	KU681137.1	NA1
Orthopneumovirus	*Orthopneumovirus hominis*	Respiratory syncytial virus A	MK634161.1	NA1
Orthopneumovirus	*Orthopneumovirus hominis*	Respiratory syncytial virus A	JX256960.1	NA2
Orthopneumovirus	*Orthopneumovirus hominis*	Respiratory syncytial virus A	AB603443.1	NA2
Orthopneumovirus	*Orthopneumovirus hominis*	Respiratory syncytial virus A	AB603445.1	NA2
Orthopneumovirus	*Orthopneumovirus hominis*	Respiratory syncytial virus A	KC297260.1	NA3
Orthopneumovirus	*Orthopneumovirus hominis*	Respiratory syncytial virus A	KC297277.1	NA3
Orthopneumovirus	*Orthopneumovirus hominis*	Respiratory syncytial virus A	KC297292.1	NA3
Orthopneumovirus	*Orthopneumovirus hominis*	Respiratory syncytial virus A	KC297324.1	NA4
Orthopneumovirus	*Orthopneumovirus hominis*	Respiratory syncytial virus A	KC297381.1	NA4
Orthopneumovirus	*Orthopneumovirus hominis*	Respiratory syncytial virus A	JN257694.1	ON1
Orthopneumovirus	*Orthopneumovirus hominis*	Respiratory syncytial virus A	KF300973.1	ON1
Orthopneumovirus	*Orthopneumovirus hominis*	Respiratory syncytial virus A	KP792361.1	ON1
Orthopneumovirus	*Orthopneumovirus hominis*	Respiratory syncytial virus A	KP792362.1	ON1
Orthopneumovirus	*Orthopneumovirus hominis*	Respiratory syncytial virus A	KP792365.1	ON1
Orthopneumovirus	*Orthopneumovirus hominis*	Respiratory syncytial virus A	KP792370.1	ON1
Orthopneumovirus	*Orthopneumovirus hominis*	Respiratory syncytial virus A	KP792373.1	ON1
Orthopneumovirus	*Orthopneumovirus hominis*	Respiratory syncytial virus A	KP792374.1	ON1
Orthopneumovirus	*Orthopneumovirus hominis*	Respiratory syncytial virus A	KP792375.1	ON1
Orthopneumovirus	*Orthopneumovirus hominis*	Respiratory syncytial virus A	AB808757.1	ON1
Orthopneumovirus	*Orthopneumovirus hominis*	Respiratory syncytial virus A	JN257693.1	ON1
Orthopneumovirus	*Orthopneumovirus hominis*	Respiratory syncytial virus A	JN257694.1	ON1
Orthopneumovirus	*Orthopneumovirus hominis*	Respiratory syncytial virus A	JX912364.1	ON1
Orthopneumovirus	*Orthopneumovirus hominis*	Respiratory syncytial virus A	KF587987.1	ON1
Orthopneumovirus	*Orthopneumovirus hominis*	Respiratory syncytial virus A	KJ710405.1	ON1
Orthopneumovirus	*Orthopneumovirus hominis*	Respiratory syncytial virus A	KT326802.1	ON1

Orthopneumovirus	*Orthopneumovirus hominis*	Respiratory syncytial virus A	KT326812.1	ON1
Orthopneumovirus	*Orthopneumovirus hominis*	Respiratory syncytial virus A	KT781387.1	ON1
Orthopneumovirus	*Orthopneumovirus hominis*	Respiratory syncytial virus A	LC037732.1	ON1
Orthopneumovirus	*Orthopneumovirus hominis*	Respiratory syncytial virus A	MG971431.1	ON1
Orthopneumovirus	*Orthopneumovirus hominis*	Respiratory syncytial virus A	MH129254.1	ON1
Orthopneumovirus	*Orthopneumovirus hominis*	Respiratory syncytial virus A	MH760648.1	ON1
Orthopneumovirus	*Orthopneumovirus hominis*	Respiratory syncytial virus A	MK634186.1	ON1
Orthopneumovirus	*Orthopneumovirus hominis*	Respiratory syncytial virus A	MK634197.1	ON1
Orthopneumovirus	*Orthopneumovirus hominis*	Respiratory syncytial virus A	MK634204.1	ON1
Orthopneumovirus	*Orthopneumovirus hominis*	Respiratory syncytial virus A	MK634222.1	ON1
Orthopneumovirus	*Orthopneumovirus hominis*	Respiratory syncytial virus A	MK634223.1	ON1
Orthopneumovirus	*Orthopneumovirus hominis*	Respiratory syncytial virus A	MK634232.1	ON1
Orthopneumovirus	*Orthopneumovirus hominis*	Respiratory syncytial virus A	MK634234.1	ON1
Orthopneumovirus	*Orthopneumovirus hominis*	Respiratory syncytial virus A	MK634235.1	ON1
Orthopneumovirus	*Orthopneumovirus hominis*	Respiratory syncytial virus A	MK634245.1	ON1
Orthopneumovirus	*Orthopneumovirus hominis*	Respiratory syncytial virus A	MK634246.1	ON1
Orthopneumovirus	*Orthopneumovirus hominis*	Respiratory syncytial virus A	MK634258.1	ON1
Orthopneumovirus	*Orthopneumovirus hominis*	Respiratory syncytial virus A	MK634259.1	ON1
Orthopneumovirus	*Orthopneumovirus hominis*	Respiratory syncytial virus A	MK634282.1	ON1
Orthopneumovirus	*Orthopneumovirus hominis*	Respiratory syncytial virus A	MN122462.1	ON1
Orthopneumovirus	*Orthopneumovirus hominis*	Respiratory syncytial virus A	AY911262.1	PRO
Orthopneumovirus	*Orthopneumovirus hominis*	Respiratory syncytial virus A	M17212.1	PRO
Orthopneumovirus	*Orthopneumovirus hominis*	Respiratory syncytial virus A	AF348807.1	SAA1
Orthopneumovirus	*Orthopneumovirus hominis*	Respiratory syncytial virus A	AF348808.1	SAA1
Orthopneumovirus	*Orthopneumovirus hominis*	Respiratory syncytial virus A	MF173101.1	GA5
Orthopneumovirus	*Orthopneumovirus hominis*	Respiratory syncytial virus A	MF173102.1	GA2
Orthopneumovirus	*Orthopneumovirus hominis*	Respiratory syncytial virus A	MF173103.1	GA2
Orthopneumovirus	*Orthopneumovirus hominis*	Respiratory syncytial virus A	MF173104.1	GA2
Orthopneumovirus	*Orthopneumovirus hominis*	Respiratory syncytial virus A	MF173105.1	GA2
Orthopneumovirus	*Orthopneumovirus hominis*	Respiratory syncytial virus A	MF372390.1	GA2
Orthopneumovirus	*Orthopneumovirus hominis*	Respiratory syncytial virus A	MF372391.1	GA2

Orthopneumovirus	*Orthopneumovirus hominis*	Respiratory syncytial virus A	MF372392.1	GA2
Orthopneumovirus	*Orthopneumovirus hominis*	Respiratory syncytial virus A	MF372393.1	GA5
Orthopneumovirus	*Orthopneumovirus hominis*	Respiratory syncytial virus A	MF372394.1	GA2
Orthopneumovirus	*Orthopneumovirus hominis*	Respiratory syncytial virus A	MF372395.1	GA2
Orthopneumovirus	*Orthopneumovirus hominis*	Respiratory syncytial virus A	MF372396.1	GA2
Orthopneumovirus	*Orthopneumovirus hominis*	Respiratory syncytial virus A	MF372397.1	GA2
Orthopneumovirus	*Orthopneumovirus hominis*	Respiratory syncytial virus A	MF372398.1	GA2
Orthopneumovirus	*Orthopneumovirus hominis*	Respiratory syncytial virus A	MF372399.1	GA5
Orthopneumovirus	*Orthopneumovirus hominis*	Respiratory syncytial virus A	MF372400.1	GA2
Orthopneumovirus	*Orthopneumovirus hominis*	Respiratory syncytial virus A	MF372401.1	GA5
Orthopneumovirus	*Orthopneumovirus hominis*	Respiratory syncytial virus A	MF372402.1	GA5
Orthopneumovirus	*Orthopneumovirus hominis*	Respiratory syncytial virus A	MF372403.1	GA2
Orthopneumovirus	*Orthopneumovirus hominis*	Respiratory syncytial virus A	MF372404.1	GA5
Orthopneumovirus	*Orthopneumovirus hominis*	Respiratory syncytial virus A	MF372405.1	GA5
Orthopneumovirus	*Orthopneumovirus hominis*	Respiratory syncytial virus A	MF372414.1	GA5
Orthopneumovirus	*Orthopneumovirus hominis*	Respiratory syncytial virus B	AY333364.1	BA1
Orthopneumovirus	*Orthopneumovirus hominis*	Respiratory syncytial virus B	DQ227363.1	BA1
Orthopneumovirus	*Orthopneumovirus hominis*	Respiratory syncytial virus B	DQ227364.1	BA1
Orthopneumovirus	*Orthopneumovirus hominis*	Respiratory syncytial virus B	DQ227368.1	BA1
Orthopneumovirus	*Orthopneumovirus hominis*	Respiratory syncytial virus B	DQ227373.1	BA1
Orthopneumovirus	*Orthopneumovirus hominis*	Respiratory syncytial virus B	DQ227374.1	BA1
Orthopneumovirus	*Orthopneumovirus hominis*	Respiratory syncytial virus B	HM459883.1	BA10
Orthopneumovirus	*Orthopneumovirus hominis*	Respiratory syncytial virus B	HM459884.1	BA10
Orthopneumovirus	*Orthopneumovirus hominis*	Respiratory syncytial virus B	HM459886.1	BA10
Orthopneumovirus	*Orthopneumovirus hominis*	Respiratory syncytial virus B	HM459888.1	BA10
Orthopneumovirus	*Orthopneumovirus hominis*	Respiratory syncytial virus B	HM459890.1	BA10
Orthopneumovirus	*Orthopneumovirus hominis*	Respiratory syncytial virus B	HM459891.1	BA10
Orthopneumovirus	*Orthopneumovirus hominis*	Respiratory syncytial virus B	KC297426.1	BA10
Orthopneumovirus	*Orthopneumovirus hominis*	Respiratory syncytial virus B	KP336523.1	BA11
Orthopneumovirus	*Orthopneumovirus hominis*	Respiratory syncytial virus B	KP336524.1	BA11
Orthopneumovirus	*Orthopneumovirus hominis*	Respiratory syncytial virus B	KP336525.1	BA11

Orthopneumovirus	*Orthopneumovirus hominis*	Respiratory syncytial virus B	KP336526.1	BA11
Orthopneumovirus	*Orthopneumovirus hominis*	Respiratory syncytial virus B	KP336527.1	BA11
Orthopneumovirus	*Orthopneumovirus hominis*	Respiratory syncytial virus B	KP336528.1	BA11
Orthopneumovirus	*Orthopneumovirus hominis*	Respiratory syncytial virus B	KP336530.1	BA11
Orthopneumovirus	*Orthopneumovirus hominis*	Respiratory syncytial virus B	KP336531.1	BA11
Orthopneumovirus	*Orthopneumovirus hominis*	Respiratory syncytial virus B	KP336532.1	BA11
Orthopneumovirus	*Orthopneumovirus hominis*	Respiratory syncytial virus B	KP336533.1	BA11
Orthopneumovirus	*Orthopneumovirus hominis*	Respiratory syncytial virus B	KP336534.1	BA11
Orthopneumovirus	*Orthopneumovirus hominis*	Respiratory syncytial virus B	KP336535.1	BA11
Orthopneumovirus	*Orthopneumovirus hominis*	Respiratory syncytial virus B	KP336536.1	BA11
Orthopneumovirus	*Orthopneumovirus hominis*	Respiratory syncytial virus B	KP336537.1	BA11
Orthopneumovirus	*Orthopneumovirus hominis*	Respiratory syncytial virus B	KP336538.1	BA11
Orthopneumovirus	*Orthopneumovirus hominis*	Respiratory syncytial virus B	KP336540.1	BA11
Orthopneumovirus	*Orthopneumovirus hominis*	Respiratory syncytial virus B	KP336541.1	BA11
Orthopneumovirus	*Orthopneumovirus hominis*	Respiratory syncytial virus B	KP336542.1	BA11
Orthopneumovirus	*Orthopneumovirus hominis*	Respiratory syncytial virus B	KP336543.1	BA11
Orthopneumovirus	*Orthopneumovirus hominis*	Respiratory syncytial virus B	KP336544.1	BA11
Orthopneumovirus	*Orthopneumovirus hominis*	Respiratory syncytial virus B	KP336545.1	BA11
Orthopneumovirus	*Orthopneumovirus hominis*	Respiratory syncytial virus B	JX256976.1	BA12
Orthopneumovirus	*Orthopneumovirus hominis*	Respiratory syncytial virus B	JX256977.1	BA12
Orthopneumovirus	*Orthopneumovirus hominis*	Respiratory syncytial virus B	KF246585.1	BA12
Orthopneumovirus	*Orthopneumovirus hominis*	Respiratory syncytial virus B	KF246586.1	BA12
Orthopneumovirus	*Orthopneumovirus hominis*	Respiratory syncytial virus B	KX262619.1	BA13
Orthopneumovirus	*Orthopneumovirus hominis*	Respiratory syncytial virus B	KX262621.1	BA13
Orthopneumovirus	*Orthopneumovirus hominis*	Respiratory syncytial virus B	KX262625.1	BA13
Orthopneumovirus	*Orthopneumovirus hominis*	Respiratory syncytial virus B	KX262638.1	BA13
Orthopneumovirus	*Orthopneumovirus hominis*	Respiratory syncytial virus B	KF300952.2	BA14
Orthopneumovirus	*Orthopneumovirus hominis*	Respiratory syncytial virus B	KF300953.2	BA14
Orthopneumovirus	*Orthopneumovirus hominis*	Respiratory syncytial virus B	KF300954.2	BA14
Orthopneumovirus	*Orthopneumovirus hominis*	Respiratory syncytial virus B	KF300955.2	BA14
Orthopneumovirus	*Orthopneumovirus hominis*	Respiratory syncytial virus B	KF300957.2	BA14

Orthopneumovirus	*Orthopneumovirus hominis*	Respiratory syncytial virus B	KF300958.2	BA14
Orthopneumovirus	*Orthopneumovirus hominis*	Respiratory syncytial virus B	KF300959.2	BA14
Orthopneumovirus	*Orthopneumovirus hominis*	Respiratory syncytial virus B	KF300960.2	BA14
Orthopneumovirus	*Orthopneumovirus hominis*	Respiratory syncytial virus B	KF300970.2	BA14
Orthopneumovirus	*Orthopneumovirus hominis*	Respiratory syncytial virus B	KX371866.1	BA14
Orthopneumovirus	*Orthopneumovirus hominis*	Respiratory syncytial virus B	KX371868.1	BA14
Orthopneumovirus	*Orthopneumovirus hominis*	Respiratory syncytial virus B	AB175821.1	BA2
Orthopneumovirus	*Orthopneumovirus hominis*	Respiratory syncytial virus B	AY751119.1	BA2
Orthopneumovirus	*Orthopneumovirus hominis*	Respiratory syncytial virus B	AY751121.1	BA2
Orthopneumovirus	*Orthopneumovirus hominis*	Respiratory syncytial virus B	AY751122.1	BA2
Orthopneumovirus	*Orthopneumovirus hominis*	Respiratory syncytial virus B	AY751123.1	BA2
Orthopneumovirus	*Orthopneumovirus hominis*	Respiratory syncytial virus B	DQ227377.1	BA2
Orthopneumovirus	*Orthopneumovirus hominis*	Respiratory syncytial virus B	DQ227389.1	BA2
Orthopneumovirus	*Orthopneumovirus hominis*	Respiratory syncytial virus B	DQ227393.1	BA2
Orthopneumovirus	*Orthopneumovirus hominis*	Respiratory syncytial virus B	DQ227370.1	BA3
Orthopneumovirus	*Orthopneumovirus hominis*	Respiratory syncytial virus B	DQ227375.1	BA3
Orthopneumovirus	*Orthopneumovirus hominis*	Respiratory syncytial virus B	DQ227397.1	BA3
Orthopneumovirus	*Orthopneumovirus hominis*	Respiratory syncytial virus B	DQ227403.1	BA3
Orthopneumovirus	*Orthopneumovirus hominis*	Respiratory syncytial virus B	DQ227396.1	BA4
Orthopneumovirus	*Orthopneumovirus hominis*	Respiratory syncytial virus B	DQ227407.1	BA4
Orthopneumovirus	*Orthopneumovirus hominis*	Respiratory syncytial virus B	DQ227408.1	BA4
Orthopneumovirus	*Orthopneumovirus hominis*	Respiratory syncytial virus B	HM459858.1	BA4
Orthopneumovirus	*Orthopneumovirus hominis*	Respiratory syncytial virus B	HM459860.1	BA4
Orthopneumovirus	*Orthopneumovirus hominis*	Respiratory syncytial virus B	HM459861.1	BA4
Orthopneumovirus	*Orthopneumovirus hominis*	Respiratory syncytial virus B	HM459863.1	BA4
Orthopneumovirus	*Orthopneumovirus hominis*	Respiratory syncytial virus B	AB175819.1	BA5
Orthopneumovirus	*Orthopneumovirus hominis*	Respiratory syncytial virus B	AB175820.1	BA5
Orthopneumovirus	*Orthopneumovirus hominis*	Respiratory syncytial virus B	AB603480.1	BA5
Orthopneumovirus	*Orthopneumovirus hominis*	Respiratory syncytial virus B	AB603482.1	BA5
Orthopneumovirus	*Orthopneumovirus hominis*	Respiratory syncytial virus B	AB603483.1	BA5
Orthopneumovirus	*Orthopneumovirus hominis*	Respiratory syncytial virus B	AB603484.1	BA5

Orthopneumovirus	*Orthopneumovirus hominis*	Respiratory syncytial virus B	AY751105.1	BA6
Orthopneumovirus	*Orthopneumovirus hominis*	Respiratory syncytial virus B	AY751111.1	BA6
Orthopneumovirus	*Orthopneumovirus hominis*	Respiratory syncytial virus B	AY751116.1	BA6
Orthopneumovirus	*Orthopneumovirus hominis*	Respiratory syncytial virus B	AY751117.1	BA6
Orthopneumovirus	*Orthopneumovirus hominis*	Respiratory syncytial virus B	AB470481.1	BA7
Orthopneumovirus	*Orthopneumovirus hominis*	Respiratory syncytial virus B	AB603476.1	BA7
Orthopneumovirus	*Orthopneumovirus hominis*	Respiratory syncytial virus B	AY751087.1	BA7
Orthopneumovirus	*Orthopneumovirus hominis*	Respiratory syncytial virus B	HM459864.1	BA7
Orthopneumovirus	*Orthopneumovirus hominis*	Respiratory syncytial virus B	HM459865.1	BA7
Orthopneumovirus	*Orthopneumovirus hominis*	Respiratory syncytial virus B	HM459866.1	BA7
Orthopneumovirus	*Orthopneumovirus hominis*	Respiratory syncytial virus B	HM459867.1	BA7
Orthopneumovirus	*Orthopneumovirus hominis*	Respiratory syncytial virus B	HM459868.1	BA7
Orthopneumovirus	*Orthopneumovirus hominis*	Respiratory syncytial virus B	HM459870.1	BA7
Orthopneumovirus	*Orthopneumovirus hominis*	Respiratory syncytial virus B	AB470482.1	BA8
Orthopneumovirus	*Orthopneumovirus hominis*	Respiratory syncytial virus B	AB603477.1	BA8
Orthopneumovirus	*Orthopneumovirus hominis*	Respiratory syncytial virus B	HM459871.1	BA8
Orthopneumovirus	*Orthopneumovirus hominis*	Respiratory syncytial virus B	HM459872.1	BA8
Orthopneumovirus	*Orthopneumovirus hominis*	Respiratory syncytial virus B	HM459875.1	BA8
Orthopneumovirus	*Orthopneumovirus hominis*	Respiratory syncytial virus B	AB603467.1	BA9
Orthopneumovirus	*Orthopneumovirus hominis*	Respiratory syncytial virus B	AB603469.1	BA9
Orthopneumovirus	*Orthopneumovirus hominis*	Respiratory syncytial virus B	AB603470.1	BA9
Orthopneumovirus	*Orthopneumovirus hominis*	Respiratory syncytial virus B	EU635867.1	BA9
Orthopneumovirus	*Orthopneumovirus hominis*	Respiratory syncytial virus B	HM459878.1	BA9
Orthopneumovirus	*Orthopneumovirus hominis*	Respiratory syncytial virus B	HM459880.1	BA9
Orthopneumovirus	*Orthopneumovirus hominis*	Respiratory syncytial virus B	HM459881.1	BA9
Orthopneumovirus	*Orthopneumovirus hominis*	Respiratory syncytial virus B	HM459882.1	BA9
Orthopneumovirus	*Orthopneumovirus hominis*	Respiratory syncytial virus B	KF246607.1	BA9
Orthopneumovirus	*Orthopneumovirus hominis*	Respiratory syncytial virus B	KF246624.1	BA9
Orthopneumovirus	*Orthopneumovirus hominis*	Respiratory syncytial virus B	KF246629.1	BA9
Orthopneumovirus	*Orthopneumovirus hominis*	Respiratory syncytial virus B	HM459879.1	BA9
Orthopneumovirus	*Orthopneumovirus hominis*	Respiratory syncytial virus B	KC297456.1	BA/C

Orthopneumovirus	*Orthopneumovirus hominis*	Respiratory syncytial virus B	KC297486.1	BA/C
Orthopneumovirus	*Orthopneumovirus hominis*	Respiratory syncytial virus B	KU254641.1	BA/CCA
Orthopneumovirus	*Orthopneumovirus hominis*	Respiratory syncytial virus B	KU254642.1	BA/CCA
Orthopneumovirus	*Orthopneumovirus hominis*	Respiratory syncytial virus B	KU254638.1	BA/CCB
Orthopneumovirus	*Orthopneumovirus hominis*	Respiratory syncytial virus B	KU254643.1	BA/CCB
Orthopneumovirus	*Orthopneumovirus hominis*	Respiratory syncytial virus B	KC297428.1	CB1
Orthopneumovirus	*Orthopneumovirus hominis*	Respiratory syncytial virus B	AF013254.1	GB1
Orthopneumovirus	*Orthopneumovirus hominis*	Respiratory syncytial virus B	AF065250.1	GB1
Orthopneumovirus	*Orthopneumovirus hominis*	Respiratory syncytial virus B	AY751256.1	GB1
Orthopneumovirus	*Orthopneumovirus hominis*	Respiratory syncytial virus B	M73540.1	GB1
Orthopneumovirus	*Orthopneumovirus hominis*	Respiratory syncytial virus B	M73541.1	GB1
Orthopneumovirus	*Orthopneumovirus hominis*	Respiratory syncytial virus B	M73542.1	GB1
Orthopneumovirus	*Orthopneumovirus hominis*	Respiratory syncytial virus B	AY751174.1	GB12
Orthopneumovirus	*Orthopneumovirus hominis*	Respiratory syncytial virus B	DQ171867.1	GB13
Orthopneumovirus	*Orthopneumovirus hominis*	Respiratory syncytial virus B	DQ171878.1	GB13
Orthopneumovirus	*Orthopneumovirus hominis*	Respiratory syncytial virus B	AF065251.1	GB2
Orthopneumovirus	*Orthopneumovirus hominis*	Respiratory syncytial virus B	DQ171849.1	GB2
Orthopneumovirus	*Orthopneumovirus hominis*	Respiratory syncytial virus B	DQ171858.1	GB2
Orthopneumovirus	*Orthopneumovirus hominis*	Respiratory syncytial virus B	AF233929.1	GB3
Orthopneumovirus	*Orthopneumovirus hominis*	Respiratory syncytial virus B	AF233932.1	GB3
Orthopneumovirus	*Orthopneumovirus hominis*	Respiratory syncytial virus B	AF233933.1	GB3
Orthopneumovirus	*Orthopneumovirus hominis*	Respiratory syncytial virus B	AF348817.1	GB3
Orthopneumovirus	*Orthopneumovirus hominis*	Respiratory syncytial virus B	AF233924.1	GB4
Orthopneumovirus	*Orthopneumovirus hominis*	Respiratory syncytial virus B	AF233928.1	GB4
Orthopneumovirus	*Orthopneumovirus hominis*	Respiratory syncytial virus B	AF233931.1	GB4
Orthopneumovirus	*Orthopneumovirus hominis*	Respiratory syncytial virus B	AF348824.1	GB4
Orthopneumovirus	*Orthopneumovirus hominis*	Respiratory syncytial virus B	AY672691.1	GB4
Orthopneumovirus	*Orthopneumovirus hominis*	Respiratory syncytial virus B	AY672698.1	GB4
Orthopneumovirus	*Orthopneumovirus hominis*	Respiratory syncytial virus B	AY751280.1	GB5
Orthopneumovirus	*Orthopneumovirus hominis*	Respiratory syncytial virus B	AY751281.1	GB5
Orthopneumovirus	*Orthopneumovirus hominis*	Respiratory syncytial virus B	AY751237.1	GB6

Orthopneumovirus	*Orthopneumovirus hominis*	Respiratory syncytial virus B	AY751239.1	GB6
Orthopneumovirus	*Orthopneumovirus hominis*	Respiratory syncytial virus B	AY751241.1	GB6
Orthopneumovirus	*Orthopneumovirus hominis*	Respiratory syncytial virus B	AB161386.1	JAB1
Orthopneumovirus	*Orthopneumovirus hominis*	Respiratory syncytial virus B	AB161387.1	JAB1
Orthopneumovirus	*Orthopneumovirus hominis*	Respiratory syncytial virus B	AB161388.1	JAB1
Orthopneumovirus	*Orthopneumovirus hominis*	Respiratory syncytial virus B	AB161389.1	JAB1
Orthopneumovirus	*Orthopneumovirus hominis*	Respiratory syncytial virus B	AB161390.1	JAB1
Orthopneumovirus	*Orthopneumovirus hominis*	Respiratory syncytial virus B	AB161391.1	JAB1
Orthopneumovirus	*Orthopneumovirus hominis*	Respiratory syncytial virus B	AB161392.1	JAB1
Orthopneumovirus	*Orthopneumovirus hominis*	Respiratory syncytial virus B	AB161395.1	JAB1
Orthopneumovirus	*Orthopneumovirus hominis*	Respiratory syncytial virus B	AB161399.1	JAB1
Orthopneumovirus	*Orthopneumovirus hominis*	Respiratory syncytial virus B	DQ171862.1	NZB1
Orthopneumovirus	*Orthopneumovirus hominis*	Respiratory syncytial virus B	DQ171863.1	NZB1
Orthopneumovirus	*Orthopneumovirus hominis*	Respiratory syncytial virus B	DQ171864.1	NZB1
Orthopneumovirus	*Orthopneumovirus hominis*	Respiratory syncytial virus B	DQ171865.1	NZB1
Orthopneumovirus	*Orthopneumovirus hominis*	Respiratory syncytial virus B	DQ171841.1	NZB2
Orthopneumovirus	*Orthopneumovirus hominis*	Respiratory syncytial virus B	DQ171842.1	NZB2
Orthopneumovirus	*Orthopneumovirus hominis*	Respiratory syncytial virus B	DQ171843.1	NZB2
Orthopneumovirus	*Orthopneumovirus hominis*	Respiratory syncytial virus B	DQ171844.1	NZB2
Orthopneumovirus	*Orthopneumovirus hominis*	Respiratory syncytial virus B	DQ171845.1	NZB2
Orthopneumovirus	*Orthopneumovirus hominis*	Respiratory syncytial virus B	DQ171846.1	NZB2
Orthopneumovirus	*Orthopneumovirus hominis*	Respiratory syncytial virus B	DQ171847.1	NZB2
Orthopneumovirus	*Orthopneumovirus hominis*	Respiratory syncytial virus B	M17213.1	PRO
Orthopneumovirus	*Orthopneumovirus hominis*	Respiratory syncytial virus B	AF348825.1	SAB1
Orthopneumovirus	*Orthopneumovirus hominis*	Respiratory syncytial virus B	AY524573.1	SAB1
Orthopneumovirus	*Orthopneumovirus hominis*	Respiratory syncytial virus B	AY660682.1	SAB1
Orthopneumovirus	*Orthopneumovirus hominis*	Respiratory syncytial virus B	JF704213.1	SAB1
Orthopneumovirus	*Orthopneumovirus hominis*	Respiratory syncytial virus B	AF309676.1	SAB2
Orthopneumovirus	*Orthopneumovirus hominis*	Respiratory syncytial virus B	AF309678.1	SAB2
Orthopneumovirus	*Orthopneumovirus hominis*	Respiratory syncytial virus B	AF348821.1	SAB2
Orthopneumovirus	*Orthopneumovirus hominis*	Respiratory syncytial virus B	AF348811.1	SAB3

Orthopneumovirus	*Orthopneumovirus hominis*	Respiratory syncytial virus B	AF348812.1	SAB3
Orthopneumovirus	*Orthopneumovirus hominis*	Respiratory syncytial virus B	AF348813.1	SAB3
Orthopneumovirus	*Orthopneumovirus hominis*	Respiratory syncytial virus B	JN119976.1	SAB4
Orthopneumovirus	*Orthopneumovirus hominis*	Respiratory syncytial virus B	JN119979.1	SAB4
Orthopneumovirus	*Orthopneumovirus hominis*	Respiratory syncytial virus B	JN119987.1	SAB4
Orthopneumovirus	*Orthopneumovirus hominis*	Respiratory syncytial virus B	JN119989.1	SAB4
Orthopneumovirus	*Orthopneumovirus hominis*	Respiratory syncytial virus B	JN120007.1	SAB4
Orthopneumovirus	*Orthopneumovirus hominis*	Respiratory syncytial virus B	KC297471.1	THB
Orthopneumovirus	*Orthopneumovirus hominis*	Respiratory syncytial virus B	KC342336.1	THB
Orthopneumovirus	*Orthopneumovirus hominis*	Respiratory syncytial virus B	KC342343.1	THB
Orthopneumovirus	*Orthopneumovirus hominis*	Respiratory syncytial virus B	AY488804.1	URU1
Orthopneumovirus	*Orthopneumovirus hominis*	Respiratory syncytial virus B	AY488805.1	URU1
Orthopneumovirus	*Orthopneumovirus hominis*	Respiratory syncytial virus B	AY333361.1	URU2
Orthopneumovirus	*Orthopneumovirus hominis*	Respiratory syncytial virus B	AY488803.1	URU2
Orthopneumovirus	*Orthopneumovirus hominis*	Respiratory syncytial virus B	MF372406.1	BA
Orthopneumovirus	*Orthopneumovirus hominis*	Respiratory syncytial virus B	MF372407.1	BA
Orthopneumovirus	*Orthopneumovirus hominis*	Respiratory syncytial virus B	MF372408.1	BA
Orthopneumovirus	*Orthopneumovirus hominis*	Respiratory syncytial virus B	MF372409.1	BA
Orthopneumovirus	*Orthopneumovirus hominis*	Respiratory syncytial virus B	MF372410.1	BA
Orthopneumovirus	*Orthopneumovirus hominis*	Respiratory syncytial virus B	MF372411.1	BA
Orthopneumovirus	*Orthopneumovirus hominis*	Respiratory syncytial virus B	MF372412.1	BA
Orthopneumovirus	*Orthopneumovirus hominis*	Respiratory syncytial virus B	MF372413.1	BA
Orthopneumovirus	*Orthopneumovirus hominis*	Respiratory syncytial virus B	MF372415.1	BA
Orthopneumovirus	*Orthopneumovirus hominis*	Respiratory syncytial virus B	MF372416.1	BA
Orthopneumovirus	*Orthopneumovirus hominis*	Respiratory syncytial virus B	MF372417.1	BA
Orthopneumovirus	*Orthopneumovirus hominis*	Respiratory syncytial virus B	MF372418.1	BA
Orthopneumovirus	*Orthopneumovirus hominis*	Respiratory syncytial virus B	MF372419.1	BA
Orthopneumovirus	*Orthopneumovirus hominis*	Respiratory syncytial virus B	MF372420.1	BA
Orthopneumovirus	*Orthopneumovirus hominis*	Respiratory syncytial virus B	MF372421.1	BA

Table S2. RV-A, RV-B and RV-C sequences used in phylogenetic analysis. This table presents the reference RV sequences retrieved from GenBank and used for phylogenetic reconstruction. The dataset includes representative genotypes of RV-A, RV-B. and RV-C. For each sequence is presented the viral genus, species, virus name, GenBank accession number and assigned genotype are provided. These reference sequences were selected for genotype assignment and phylogenetic analysis of the sequences generated in this study.

Genus	Specie	Genotype	Accession Number
Enterovirus	*Enterovirus alphacoxsackie*	A2	NC38306.1
Enterovirus	*Enterovirus alpharhino*	RV-A1	NC38311.1
Enterovirus	*Enterovirus alpharhino*	RV-A1	FJ445111.1
Enterovirus	*Enterovirus alpharhino*	RV-A10	DQ473498.1
Enterovirus	*Enterovirus alpharhino*	RV-A100	FJ445175.1
Enterovirus	*Enterovirus alpharhino*	RV-A101	GQ415051.1
Enterovirus	*Enterovirus alpharhino*	RV-A102	EF155421.1
Enterovirus	*Enterovirus alpharhino*	RV-A103	JF965515.1
Enterovirus	*Enterovirus alpharhino*	RV-A104	JN562727.1
Enterovirus	*Enterovirus alpharhino*	RV-A11	EF173414.1
Enterovirus	*Enterovirus alpharhino*	RV-A12	EF173415.1
Enterovirus	*Enterovirus alpharhino*	RV-A13	FJ445116.1
Enterovirus	*Enterovirus alpharhino*	RV-A15	DQ473493.1
Enterovirus	*Enterovirus alpharhino*	RV-A16	L24917.1
Enterovirus	*Enterovirus alpharhino*	RV-A18	FJ445118.1
Enterovirus	*Enterovirus alpharhino*	RV-A19	FJ445119.1
Enterovirus	*Enterovirus alpharhino*	RV-A1B	D00239.1
Enterovirus	*Enterovirus alpharhino*	RV-A1B	MK501734.1
Enterovirus	*Enterovirus alpharhino*	RV-A1B	OK181469.1
Enterovirus	*Enterovirus alpharhino*	RV-A2	MN749149.1
Enterovirus	*Enterovirus alpharhino*	RV-A2	OM001375.1
Enterovirus	*Enterovirus alpharhino*	RV-A2	X02316.1
Enterovirus	*Enterovirus alpharhino*	RV-A20	FJ445120.1
Enterovirus	*Enterovirus alpharhino*	RV-A20	OL638459.1
Enterovirus	*Enterovirus alpharhino*	RV-A21	FJ445121.1
Enterovirus	*Enterovirus alpharhino*	RV-A21	KM576765.1

Enterovirus	*Enterovirus alpharhino*	RV-A21	KM576766.1
Enterovirus	*Enterovirus alpharhino*	RV-A21	LC699421.1
Enterovirus	*Enterovirus alpharhino*	RV-A21	OK649399.1
Enterovirus	*Enterovirus alpharhino*	RV-A21	ON729333.1
Enterovirus	*Enterovirus alpharhino*	RV-A22	FJ445122.1
Enterovirus	*Enterovirus alpharhino*	RV-A22	KY369885.1
Enterovirus	*Enterovirus alpharhino*	RV-A22	ON311254.1
Enterovirus	*Enterovirus alpharhino*	RV-A22	OP342736.1
Enterovirus	*Enterovirus alpharhino*	RV-A23	DQ473497.1
Enterovirus	*Enterovirus alpharhino*	RV-A23	OL638422.1
Enterovirus	*Enterovirus alpharhino*	RV-A23	ON311223.1
Enterovirus	*Enterovirus alpharhino*	RV-A24	EF173416.1
Enterovirus	*Enterovirus alpharhino*	RV-A24	LC699415.1
Enterovirus	*Enterovirus alpharhino*	RV-A24	OL638419.1
Enterovirus	*Enterovirus alpharhino*	RV-A25	FJ445123.1
Enterovirus	*Enterovirus alpharhino*	RV-A25	OL638418.1
Enterovirus	*Enterovirus alpharhino*	RV-A25	ON311219.1
Enterovirus	*Enterovirus alpharhino*	RV-A28	DQ473508.1
Enterovirus	*Enterovirus alpharhino*	RV-A28	OL133753.1
Enterovirus	*Enterovirus alpharhino*	RV-A28	ON881123.1
Enterovirus	*Enterovirus alpharhino*	RV-A28	OP342734.1
Enterovirus	*Enterovirus alpharhino*	RV-A29	FJ445125.1
Enterovirus	*Enterovirus alpharhino*	RV-A29	LC817388.1
Enterovirus	*Enterovirus alpharhino*	RV-A29	OL638456.1
Enterovirus	*Enterovirus alpharhino*	RV-A30	DQ473512.1
Enterovirus	*Enterovirus alpharhino*	RV-A30	OL638454.1
Enterovirus	*Enterovirus alpharhino*	RV-A30	OP342733.1
Enterovirus	*Enterovirus alpharhino*	RV-A31	FJ445126.1
Enterovirus	*Enterovirus alpharhino*	RV-A31	KY369884.1
Enterovirus	*Enterovirus alpharhino*	RV-A31	OL638462.1
Enterovirus	*Enterovirus alpharhino*	RV-A32	FJ445127.1

Enterovirus	*Enterovirus alpharhino*	RV-A33	FJ445128.1
Enterovirus	*Enterovirus alpharhino*	RV-A33	OP342732.1
Enterovirus	*Enterovirus alpharhino*	RV-A34	DQ473501.1
Enterovirus	*Enterovirus alpharhino*	RV-A34	ON311270.1
Enterovirus	*Enterovirus alpharhino*	RV-A34	OP342731.1
Enterovirus	*Enterovirus alpharhino*	RV-A36	DQ473505.1
Enterovirus	*Enterovirus alpharhino*	RV-A38	DQ473495.1
Enterovirus	*Enterovirus alpharhino*	RV-A38	OP342729.1
Enterovirus	*Enterovirus alpharhino*	RV-A39	AY751783.1
Enterovirus	*Enterovirus alpharhino*	RV-A39	MZ629137.1
Enterovirus	*Enterovirus alpharhino*	RV-A39	ON311218.1
Enterovirus	*Enterovirus alpharhino*	RV-A40	FJ445129.1
Enterovirus	*Enterovirus alpharhino*	RV-A40	LC720413.1
Enterovirus	*Enterovirus alpharhino*	RV-A41	DQ473491.1
Enterovirus	*Enterovirus alpharhino*	RV-A41	MN369037.1
Enterovirus	*Enterovirus alpharhino*	RV-A43	FJ445131.1
Enterovirus	*Enterovirus alpharhino*	RV-A45	FJ445132.1
Enterovirus	*Enterovirus alpharhino*	RV-A45	OP342727.1
Enterovirus	*Enterovirus alpharhino*	RV-A46	DQ473506.1
Enterovirus	*Enterovirus alpharhino*	RV-A46	OK254831.1
Enterovirus	*Enterovirus alpharhino*	RV-A47	FJ445133.1
Enterovirus	*Enterovirus alpharhino*	RV-A47	OM001356.1
Enterovirus	*Enterovirus alpharhino*	RV-A49	DQ473496.1
Enterovirus	*Enterovirus alpharhino*	RV-A49	OP342726.1
Enterovirus	*Enterovirus alpharhino*	RV-A50	FJ445135.1
Enterovirus	*Enterovirus alpharhino*	RV-A51	FJ445136.1
Enterovirus	*Enterovirus alpharhino*	RV-A51	OP342725.1
Enterovirus	*Enterovirus alpharhino*	RV-A53	DQ473507.1
Enterovirus	*Enterovirus alpharhino*	RV-A53	OK017946.1
Enterovirus	*Enterovirus alpharhino*	RV-A53	OP342724.1
Enterovirus	*Enterovirus alpharhino*	RV-A54	FJ445138.1

Enterovirus	*Enterovirus alpharhino*	RV-A54	OL961530.1
Enterovirus	*Enterovirus alpharhino*	RV-A54	OP342722.1
Enterovirus	*Enterovirus alpharhino*	RV-A55	DQ473511.1
Enterovirus	*Enterovirus alpharhino*	RV-A56	FJ445140.1
Enterovirus	*Enterovirus alpharhino*	RV-A56	OP342720.1
Enterovirus	*Enterovirus alpharhino*	RV-A56	OQ116582.1
Enterovirus	*Enterovirus alpharhino*	RV-A57	FJ445141.1
Enterovirus	*Enterovirus alpharhino*	RV-A58	FJ445142.1
Enterovirus	*Enterovirus alpharhino*	RV-A58	OL961521.1
Enterovirus	*Enterovirus alpharhino*	RV-A58	OP342718.1
Enterovirus	*Enterovirus alpharhino*	RV-A59	DQ473500.1
Enterovirus	*Enterovirus alpharhino*	RV-A59	ON729338.1
Enterovirus	*Enterovirus alpharhino*	RV-A60	FJ445143.1
Enterovirus	*Enterovirus alpharhino*	RV-A60	ON729329.1
Enterovirus	*Enterovirus alpharhino*	RV-A60	OP342717.1
Enterovirus	*Enterovirus alpharhino*	RV-A61	FJ445144.1
Enterovirus	*Enterovirus alpharhino*	RV-A61	OL961529.1
Enterovirus	*Enterovirus alpharhino*	RV-A61	ON881121.1
Enterovirus	*Enterovirus alpharhino*	RV-A62	FJ445145.1
Enterovirus	*Enterovirus alpharhino*	RV-A63	FJ445146.1
Enterovirus	*Enterovirus alpharhino*	RV-A64	EF173417.1
Enterovirus	*Enterovirus alpharhino*	RV-A64	FJ445181.1
Enterovirus	*Enterovirus alpharhino*	RV-A65	FJ445147.1
Enterovirus	*Enterovirus alpharhino*	RV-A66	FJ445148.1
Enterovirus	*Enterovirus alpharhino*	RV-A66	OK649378.1
Enterovirus	*Enterovirus alpharhino*	RV-A67	FJ445149.1
Enterovirus	*Enterovirus alpharhino*	RV-A67	PP411923.1
Enterovirus	*Enterovirus alpharhino*	RV-A68	FJ445150.1
Enterovirus	*Enterovirus alpharhino*	RV-A68	KY369887.1
Enterovirus	*Enterovirus alpharhino*	RV-A7	DQ473503.1
Enterovirus	*Enterovirus alpharhino*	RV-A7	ON311264.1

Enterovirus	*Enterovirus alpharhino*	RV-A71	FJ445152.1
Enterovirus	*Enterovirus alpharhino*	RV-A73	DQ473492.1
Enterovirus	*Enterovirus alpharhino*	RV-A73	LC720410.1
Enterovirus	*Enterovirus alpharhino*	RV-A74	DQ473494.1
Enterovirus	*Enterovirus alpharhino*	RV-A75	DQ473510.1
Enterovirus	*Enterovirus alpharhino*	RV-A76	DQ473502.1
Enterovirus	*Enterovirus alpharhino*	RV-A76	OP342714.1
Enterovirus	*Enterovirus alpharhino*	RV-A77	FJ445154.1
Enterovirus	*Enterovirus alpharhino*	RV-A77	PP411922.1
Enterovirus	*Enterovirus alpharhino*	RV-A78	EF173418.1
Enterovirus	*Enterovirus alpharhino*	RV-A78	LC699414.1
Enterovirus	*Enterovirus alpharhino*	RV-A78	OK254871.1
Enterovirus	*Enterovirus alpharhino*	RV-A8	FJ445113.1
Enterovirus	*Enterovirus alpharhino*	RV-A80	FJ445156.1
Enterovirus	*Enterovirus alpharhino*	RV-A80	LC817387.1
Enterovirus	*Enterovirus alpharhino*	RV-A80	OL638424.1
Enterovirus	*Enterovirus alpharhino*	RV-A80	OP342713.1
Enterovirus	*Enterovirus alpharhino*	RV-A81	FJ445157.1
Enterovirus	*Enterovirus alpharhino*	RV-A82	DQ473509.1
Enterovirus	*Enterovirus alpharhino*	RV-A85	FJ445163.1
Enterovirus	*Enterovirus alpharhino*	RV-A85	ON729334.1
Enterovirus	*Enterovirus alpharhino*	RV-A88	DQ473504.1
Enterovirus	*Enterovirus alpharhino*	RV-A88	LC699419.1
Enterovirus	*Enterovirus alpharhino*	RV-A89	M16248.1
Enterovirus	*Enterovirus alpharhino*	RV-A9	FJ445177.1
Enterovirus	*Enterovirus alpharhino*	RV-A9	OL133739.1
Enterovirus	*Enterovirus alpharhino*	RV-A9	ON881120.1
Enterovirus	*Enterovirus alpharhino*	RV-A90	FJ445167.1
Enterovirus	*Enterovirus alpharhino*	RV-A94	EF173419.1
Enterovirus	*Enterovirus alpharhino*	RV-A94	OK649392.1
Enterovirus	*Enterovirus alpharhino*	RV-A94	ON729330.1

Enterovirus	*Enterovirus alpharhino*	RV-A96	FJ445171.1
Enterovirus	*Enterovirus alpharhino*	RV-A98	FJ445173.1
Enterovirus	*Enterovirus betarhino*	RV-B100	HQ123444.1
Enterovirus	*Enterovirus betarhino*	RV-B100	MZ629139.1
Enterovirus	*Enterovirus betarhino*	RV-B101	JF781500.1
Enterovirus	*Enterovirus betarhino*	RV-B102	JX074053.1
Enterovirus	*Enterovirus betarhino*	RV-B103	JN614996.1
Enterovirus	*Enterovirus betarhino*	RV-B104	FJ445137.1
Enterovirus	*Enterovirus betarhino*	RV-B14	K02121.1
Enterovirus	*Enterovirus betarhino*	RV-B14	NC001490.1
Enterovirus	*Enterovirus betarhino*	RV-B17	EF173420.1
Enterovirus	*Enterovirus betarhino*	RV-B26	FJ445124.1
Enterovirus	*Enterovirus betarhino*	RV-B27	FJ445186.1
Enterovirus	*Enterovirus betarhino*	RV-B27	KY369901.1
Enterovirus	*Enterovirus betarhino*	RV-B27	ON311185.1
Enterovirus	*Enterovirus betarhino*	RV-B3	DQ473485.1
Enterovirus	*Enterovirus betarhino*	RV-B3	NC038312.1
Enterovirus	*Enterovirus betarhino*	RV-B3	OM001374.1
Enterovirus	*Enterovirus betarhino*	RV-B3	OP342704.1
Enterovirus	*Enterovirus betarhino*	RV-B3	OP342707.1
Enterovirus	*Enterovirus betarhino*	RV-B35	DQ473487.1
Enterovirus	*Enterovirus betarhino*	RV-B35	ON311181.1
Enterovirus	*Enterovirus betarhino*	RV-B37	EF173423.1
Enterovirus	*Enterovirus betarhino*	RV-B4	DQ473490.1
Enterovirus	*Enterovirus betarhino*	RV-B4	MZ629155.1
Enterovirus	*Enterovirus betarhino*	RV-B42	FJ445130.1
Enterovirus	*Enterovirus betarhino*	RV-B48	DQ473488.1
Enterovirus	*Enterovirus betarhino*	RV-B48	KY348863.1
Enterovirus	*Enterovirus betarhino*	RV-B5	FJ445112.1
Enterovirus	*Enterovirus betarhino*	RV-B52	FJ445188.1
Enterovirus	*Enterovirus betarhino*	RV-B52	ON881124.1

Enterovirus	*Enterovirus betarhino*	RV-B6	DQ473486.1
Enterovirus	*Enterovirus betarhino*	RV-B6	OL638410.1
Enterovirus	*Enterovirus betarhino*	RV-B69	FJ445151.1
Enterovirus	*Enterovirus betarhino*	RV-B70	DQ473489.1
Enterovirus	*Enterovirus betarhino*	RV-B70	OL961525.1
Enterovirus	*Enterovirus betarhino*	RV-B72	FJ445153.1
Enterovirus	*Enterovirus betarhino*	RV-B72	KY189314.1
Enterovirus	*Enterovirus betarhino*	RV-B79	FJ445155.1
Enterovirus	*Enterovirus betarhino*	RV-B79	MT512399.1
Enterovirus	*Enterovirus betarhino*	RV-B83	FJ445161.1
Enterovirus	*Enterovirus betarhino*	RV-B83	MN306025.1
Enterovirus	*Enterovirus betarhino*	RV-B84	FJ445162.1
Enterovirus	*Enterovirus betarhino*	RV-B86	FJ445164.1
Enterovirus	*Enterovirus betarhino*	RV-B86	MN369039.1
Enterovirus	*Enterovirus betarhino*	RV-B91	FJ445168.1
Enterovirus	*Enterovirus betarhino*	RV-B91	KX494871.1
Enterovirus	*Enterovirus betarhino*	RV-B91	OL961542.1
Enterovirus	*Enterovirus betarhino*	RV-B92	FJ445169.1
Enterovirus	*Enterovirus betarhino*	RV-B93	EF173425.1
Enterovirus	*Enterovirus betarhino*	RV-B97	FJ445172.1
Enterovirus	*Enterovirus betarhino*	RV-B97	KY369883.1
Enterovirus	*Enterovirus betarhino*	RV-B99	FJ445174.1
Enterovirus	*Enterovirus cerhino*	RV-C	NC009996.1
Enterovirus	*Enterovirus cerhino*	RV-C1	EF077279.1
Enterovirus	*Enterovirus cerhino*	RV-C10	GQ323774.1
Enterovirus	*Enterovirus cerhino*	RV-C11	EU840952.2
Enterovirus	*Enterovirus cerhino*	RV-C11	OQ348028.1
Enterovirus	*Enterovirus cerhino*	RV-C13	OQ116581.1
Enterovirus	*Enterovirus cerhino*	RV-C15	GU219984.1
Enterovirus	*Enterovirus cerhino*	RV-C15	OP342697.1
Enterovirus	*Enterovirus cerhino*	RV-C16	KR997882.1

Enterovirus	*Enterovirus cerhino*	RV-C17	OR726585.1
Enterovirus	*Enterovirus cerhino*	RV-C19	EU840728.1
Enterovirus	*Enterovirus cerhino*	RV-C19	MN369038.1
Enterovirus	*Enterovirus cerhino*	RV-C2	EF077280.1
Enterovirus	*Enterovirus cerhino*	RV-C2	MZ835576.1
Enterovirus	*Enterovirus cerhino*	RV-C2	ON881125.1
Enterovirus	*Enterovirus cerhino*	RV-C20	MZ427502.1
Enterovirus	*Enterovirus cerhino*	RV-C20	OL961518.1
Enterovirus	*Enterovirus cerhino*	RV-C22	KJ675507.1
Enterovirus	*Enterovirus cerhino*	RV-C22	OP342696.1
Enterovirus	*Enterovirus cerhino*	RV-C23	KJ675506.1
Enterovirus	*Enterovirus cerhino*	RV-C23	MZ540948.1
Enterovirus	*Enterovirus cerhino*	RV-C24	OP342695.1
Enterovirus	*Enterovirus cerhino*	RV-C26	OQ331223.1
Enterovirus	*Enterovirus cerhino*	RV-C27	KY369877.1
Enterovirus	*Enterovirus cerhino*	RV-C28	OK017915.1
Enterovirus	*Enterovirus cerhino*	RV-C3	EF186077.2
Enterovirus	*Enterovirus cerhino*	RV-C3	LC699425.1
Enterovirus	*Enterovirus cerhino*	RV-C32	PP314216.1
Enterovirus	*Enterovirus cerhino*	RV-C33	OM001346.1
Enterovirus	*Enterovirus cerhino*	RV-C34	KM486097.1
Enterovirus	*Enterovirus cerhino*	RV-C34	ON729335.1
Enterovirus	*Enterovirus cerhino*	RV-C35	JF436925.1
Enterovirus	*Enterovirus cerhino*	RV-C35	OP342693.1
Enterovirus	*Enterovirus cerhino*	RV-C36	OP342692.1
Enterovirus	*Enterovirus cerhino*	RV-C39	JN205461.1
Enterovirus	*Enterovirus cerhino*	RV-C4	EF582385.1
Enterovirus	*Enterovirus cerhino*	RV-C40	JN815251.1
Enterovirus	*Enterovirus cerhino*	RV-C40	OK254858.1
Enterovirus	*Enterovirus cerhino*	RV-C41	KF958311.1
Enterovirus	*Enterovirus cerhino*	RV-C41	MK279354.1

Enterovirus	*Enterovirus cerhino*	RV-C42	KJ675505.1
Enterovirus	*Enterovirus cerhino*	RV-C42	OP342691.1
Enterovirus	*Enterovirus cerhino*	RV-C43	JN815249.1
Enterovirus	*Enterovirus cerhino*	RV-C43	OK161404.1
Enterovirus	*Enterovirus cerhino*	RV-C44	OM001412.1
Enterovirus	*Enterovirus cerhino*	RV-C45	KY624849.1
Enterovirus	*Enterovirus cerhino*	RV-C47	MF806525.1
Enterovirus	*Enterovirus cerhino*	RV-C49	JN798566.1
Enterovirus	*Enterovirus cerhino*	RV-C5	EF582386.1
Enterovirus	*Enterovirus cerhino*	RV-C5	ON881137.1
Enterovirus	*Enterovirus cerhino*	RV-C50	KF688606.1
Enterovirus	*Enterovirus cerhino*	RV-C51	JF317015.1
Enterovirus	*Enterovirus cerhino*	RV-C51	ON311158.1
Enterovirus	*Enterovirus cerhino*	RV-C53	ON881138.1
Enterovirus	*Enterovirus cerhino*	RV-C54	KP282614.1
Enterovirus	*Enterovirus cerhino*	RV-C55	KR997885.1
Enterovirus	*Enterovirus cerhino*	RV-C55	LC720415.1
Enterovirus	*Enterovirus cerhino*	RV-C56	MZ629123.1
Enterovirus	*Enterovirus cerhino*	RV-C57	KP890662.1
Enterovirus	*Enterovirus cerhino*	RV-C6	EF582387.1
Enterovirus	*Enterovirus cerhino*	RV-C6	OP342702.1
Enterovirus	*Enterovirus cerhino*	RV-C7	DQ875932.2
Enterovirus	*Enterovirus cerhino*	RV-C7	OM001408.1
Enterovirus	*Enterovirus cerhino*	RV-C8	GQ223227.1
Enterovirus	*Enterovirus cerhino*	RV-C8	OL365068.1
Enterovirus	*Enterovirus cerhino*	RV-C9	GQ223228.1
Enterovirus	*Enterovirus cerhino*	RV-C9	OK649391.1

Table S3. RSV-A and RSV-B reference sequences with collection date and geographic origin used for temporal analyses. This table lists the reference RSV sequences included in the temporal and evolutionary analyses. For each sequence the viral genus, species, virus name, GenBank accession number, country of origin and year of collection are presented.

Genus	Specie	Virus Name	Accession Number	Country	Collection Date
Orthopneumovirus	*Orthopneumovirus hominis*	Respiratory syncytial virus A	OK649668.1	USA	1956
Orthopneumovirus	*Orthopneumovirus hominis*	Respiratory syncytial virus A	MG642031.1	USA	1982
Orthopneumovirus	*Orthopneumovirus hominis*	Respiratory syncytial virus A	MG642028.1	USA	1980
Orthopneumovirus	*Orthopneumovirus hominis*	Respiratory syncytial virus A	MG642024.1	USA	1981
Orthopneumovirus	*Orthopneumovirus hominis*	Respiratory syncytial virus A	MG642081.1	USA	1982
Orthopneumovirus	*Orthopneumovirus hominis*	Respiratory syncytial virus A	MG642060.1	USA	1980
Orthopneumovirus	*Orthopneumovirus hominis*	Respiratory syncytial virus A	MG642063.1	USA	1982
Orthopneumovirus	*Orthopneumovirus hominis*	Respiratory syncytial virus A	MG642038.1	USA	1985
Orthopneumovirus	*Orthopneumovirus hominis*	Respiratory syncytial virus A	MG642026.1	USA	1982
Orthopneumovirus	*Orthopneumovirus hominis*	Respiratory syncytial virus A	MG642074.1	USA	1980
Orthopneumovirus	*Orthopneumovirus hominis*	Respiratory syncytial virus A	MG642071.1	USA	1984
Orthopneumovirus	*Orthopneumovirus hominis*	Respiratory syncytial virus A	MG642056.1	USA	1982
Orthopneumovirus	*Orthopneumovirus hominis*	Respiratory syncytial virus A	MG642069.1	USA	1985
Orthopneumovirus	*Orthopneumovirus hominis*	Respiratory syncytial virus A	MG642058.1	USA	1980
Orthopneumovirus	*Orthopneumovirus hominis*	Respiratory syncytial virus A	MG642035.1	USA	1984
Orthopneumovirus	*Orthopneumovirus hominis*	Respiratory syncytial virus A	MG642079.1	USA	1982
Orthopneumovirus	*Orthopneumovirus hominis*	Respiratory syncytial virus A	MG642032.1	USA	1980
Orthopneumovirus	*Orthopneumovirus hominis*	Respiratory syncytial virus A	MG642040.1	USA	1980
Orthopneumovirus	*Orthopneumovirus hominis*	Respiratory syncytial virus A	MG642034.1	USA	1985
Orthopneumovirus	*Orthopneumovirus hominis*	Respiratory syncytial virus A	MG642052.1	USA	1994
Orthopneumovirus	*Orthopneumovirus hominis*	Respiratory syncytial virus A	MG642075.1	USA	1986
Orthopneumovirus	*Orthopneumovirus hominis*	Respiratory syncytial virus A	OK649589.1	USA	1994
Orthopneumovirus	*Orthopneumovirus hominis*	Respiratory syncytial virus A	OK649592.1	USA	1994
Orthopneumovirus	*Orthopneumovirus hominis*	Respiratory syncytial virus A	MG642080.1	USA	1994
Orthopneumovirus	*Orthopneumovirus hominis*	Respiratory syncytial virus A	OK649675.1	USA	1994
Orthopneumovirus	*Orthopneumovirus hominis*	Respiratory syncytial virus A	OK649659.1	Canada	1995

Orthopneumovirus	*Orthopneumovirus hominis*	Respiratory syncytial virus A	OK649590.1	USA	1994
Orthopneumovirus	*Orthopneumovirus hominis*	Respiratory syncytial virus A	MG642050.1	USA	1994
Orthopneumovirus	*Orthopneumovirus hominis*	Respiratory syncytial virus A	MG642083.1	USA	1987
Orthopneumovirus	*Orthopneumovirus hominis*	Respiratory syncytial virus A	MG642055.1	USA	1990
Orthopneumovirus	*Orthopneumovirus hominis*	Respiratory syncytial virus A	MG642070.1	USA	1986
Orthopneumovirus	*Orthopneumovirus hominis*	Respiratory syncytial virus A	MG642061.1	USA	1994
Orthopneumovirus	*Orthopneumovirus hominis*	Respiratory syncytial virus A	OK649676.1	USA	1993
Orthopneumovirus	*Orthopneumovirus hominis*	Respiratory syncytial virus A	OK649674.1	USA	1995
Orthopneumovirus	*Orthopneumovirus hominis*	Respiratory syncytial virus A	OK649657.1	Canada	1995
Orthopneumovirus	*Orthopneumovirus hominis*	Respiratory syncytial virus A	OK649673.1	USA	1995
Orthopneumovirus	*Orthopneumovirus hominis*	Respiratory syncytial virus A	PQ416565.1	USA	1987
Orthopneumovirus	*Orthopneumovirus hominis*	Respiratory syncytial virus A	MG642033.1	USA	1994
Orthopneumovirus	*Orthopneumovirus hominis*	Respiratory syncytial virus A	OK649669.1	USA	1994
Orthopneumovirus	*Orthopneumovirus hominis*	Respiratory syncytial virus A	KJ627659.1	USA	2003
Orthopneumovirus	*Orthopneumovirus hominis*	Respiratory syncytial virus A	KJ627679.1	USA	2001
Orthopneumovirus	*Orthopneumovirus hominis*	Respiratory syncytial virus A	KJ627714.1	USA	2003
Orthopneumovirus	*Orthopneumovirus hominis*	Respiratory syncytial virus A	OR795307.1	Germany	16/12/2002
Orthopneumovirus	*Orthopneumovirus hominis*	Respiratory syncytial virus A	OR795316.1	Germany	3/2/2005
Orthopneumovirus	*Orthopneumovirus hominis*	Respiratory syncytial virus A	KJ627690.1	USA	2001
Orthopneumovirus	*Orthopneumovirus hominis*	Respiratory syncytial virus A	KJ627682.1	USA	2001
Orthopneumovirus	*Orthopneumovirus hominis*	Respiratory syncytial virus A	KJ627684.1	USA	2001
Orthopneumovirus	*Orthopneumovirus hominis*	Respiratory syncytial virus A	KF973321.1	USA	2002
Orthopneumovirus	*Orthopneumovirus hominis*	Respiratory syncytial virus A	KJ627728.1	USA	2003
Orthopneumovirus	*Orthopneumovirus hominis*	Respiratory syncytial virus A	OR795315.1	Germany	11/1/2005
Orthopneumovirus	*Orthopneumovirus hominis*	Respiratory syncytial virus A	OR795306.1	Germany	12/12/2002
Orthopneumovirus	*Orthopneumovirus hominis*	Respiratory syncytial virus A	KJ627696.1	USA	2001
Orthopneumovirus	*Orthopneumovirus hominis*	Respiratory syncytial virus A	KF973339.1	USA	2002
Orthopneumovirus	*Orthopneumovirus hominis*	Respiratory syncytial virus A	KJ627708.1	USA	2001
Orthopneumovirus	*Orthopneumovirus hominis*	Respiratory syncytial virus A	KJ627663.1	USA	2003
Orthopneumovirus	*Orthopneumovirus hominis*	Respiratory syncytial virus A	KF973330.1	USA	2002
Orthopneumovirus	*Orthopneumovirus hominis*	Respiratory syncytial virus A	KF973336.1	USA	2002

Orthopneumovirus	*Orthopneumovirus hominis*	Respiratory syncytial virus A	OR795310.1	Germany	7/2/2004
Orthopneumovirus	*Orthopneumovirus hominis*	Respiratory syncytial virus A	OR795314.1	Germany	6/1/2005
Orthopneumovirus	*Orthopneumovirus hominis*	Respiratory syncytial virus A	OK649623.1	Brazil	2008
Orthopneumovirus	*Orthopneumovirus hominis*	Respiratory syncytial virus A	OR795318.1	Germany	27/1/2006
Orthopneumovirus	*Orthopneumovirus hominis*	Respiratory syncytial virus A	ON237286.1	Argentina	21/6/2016
Orthopneumovirus	*Orthopneumovirus hominis*	Respiratory syncytial virus A	MK109763.1	Jordan	27/1/2011
Orthopneumovirus	*Orthopneumovirus hominis*	Respiratory syncytial virus A	KY654511.1	Philippines	8/4/2013
Orthopneumovirus	*Orthopneumovirus hominis*	Respiratory syncytial virus A	MH181908.1	Kenya	13/2/2012
Orthopneumovirus	*Orthopneumovirus hominis*	Respiratory syncytial virus A	OR466353.1	USA	5/3/2013
Orthopneumovirus	*Orthopneumovirus hominis*	Respiratory syncytial virus A	PP525302.1	USA	2016
Orthopneumovirus	*Orthopneumovirus hominis*	Respiratory syncytial virus A	MH447953.1	Thailand	1/11/2013
Orthopneumovirus	*Orthopneumovirus hominis*	Respiratory syncytial virus A	MH447957.1	Thailand	1/9/2016
Orthopneumovirus	*Orthopneumovirus hominis*	Respiratory syncytial virus A	KJ643504.1	USA	2013
Orthopneumovirus	*Orthopneumovirus hominis*	Respiratory syncytial virus A	MK109787.1	Jordan	10/2/2011
Orthopneumovirus	*Orthopneumovirus hominis*	Respiratory syncytial virus A	MW582528.1	Germany	15/3/2015
Orthopneumovirus	*Orthopneumovirus hominis*	Respiratory syncytial virus A	MH181969.1	Kenya	4/12/2012
Orthopneumovirus	*Orthopneumovirus hominis*	Respiratory syncytial virus A	ON237267.1	Argentina	13/7/2015
Orthopneumovirus	*Orthopneumovirus hominis*	Respiratory syncytial virus A	OR466336.1	USA	6/11/2012
Orthopneumovirus	*Orthopneumovirus hominis*	Respiratory syncytial virus A	OK649640.1	Brazil	2009
Orthopneumovirus	*Orthopneumovirus hominis*	Respiratory syncytial virus A	OR795329.1	Germany	29/3/2010
Orthopneumovirus	*Orthopneumovirus hominis*	Respiratory syncytial virus A	KJ643507.1	USA	2013
Orthopneumovirus	*Orthopneumovirus hominis*	Respiratory syncytial virus A	KJ643543.1	USA	2013
Orthopneumovirus	*Orthopneumovirus hominis*	Respiratory syncytial virus A	MH181985.1	Kenya	17/1/2013
Orthopneumovirus	*Orthopneumovirus hominis*	Respiratory syncytial virus A	OQ848529.1	USA	1/11/2010
Orthopneumovirus	*Orthopneumovirus hominis*	Respiratory syncytial virus A	OR795322.1	Germany	5/2/2007
Orthopneumovirus	*Orthopneumovirus hominis*	Respiratory syncytial virus A	OK649630.1	Brazil	2008
Orthopneumovirus	*Orthopneumovirus hominis*	Respiratory syncytial virus A	KJ643498.1	USA	2013
Orthopneumovirus	*Orthopneumovirus hominis*	Respiratory syncytial virus A	ON237285.1	Argentina	10/6/2016
Orthopneumovirus	*Orthopneumovirus hominis*	Respiratory syncytial virus A	PP525305.1	USA	2016
Orthopneumovirus	*Orthopneumovirus hominis*	Respiratory syncytial virus A	OR466354.1	USA	7/3/2013
Orthopneumovirus	*Orthopneumovirus hominis*	Respiratory syncytial virus A	MH181933.1	Kenya	27/10/2012

Orthopneumovirus	*Orthopneumovirus hominis*	Respiratory syncytial virus A	KJ643478.1	USA	2013
Orthopneumovirus	*Orthopneumovirus hominis*	Respiratory syncytial virus A	MH181944.1	Kenya	17/11/2012
Orthopneumovirus	*Orthopneumovirus hominis*	Respiratory syncytial virus A	KY982517.1	USA	3/1/2013
Orthopneumovirus	*Orthopneumovirus hominis*	Respiratory syncytial virus A	ON237276.1	Argentina	16/5/2016
Orthopneumovirus	*Orthopneumovirus hominis*	Respiratory syncytial virus A	OR795336.1	Germany	6/3/2012
Orthopneumovirus	*Orthopneumovirus hominis*	Respiratory syncytial virus A	MH182012.1	Kenya	14/12/2014
Orthopneumovirus	*Orthopneumovirus hominis*	Respiratory syncytial virus A	MH181981.1	Kenya	10/1/2013
Orthopneumovirus	*Orthopneumovirus hominis*	Respiratory syncytial virus A	KJ643463.1	USA	2013
Orthopneumovirus	*Orthopneumovirus hominis*	Respiratory syncytial virus A	MH447952.1	Thailand	17/9/2012
Orthopneumovirus	*Orthopneumovirus hominis*	Respiratory syncytial virus A	ON237283.1	Argentina	31/5/2016
Orthopneumovirus	*Orthopneumovirus hominis*	Respiratory syncytial virus A	ON237229.1	Argentina	9/6/2014
Orthopneumovirus	*Orthopneumovirus hominis*	Respiratory syncytial virus A	KJ643550.1	USA	2013
Orthopneumovirus	*Orthopneumovirus hominis*	Respiratory syncytial virus A	MH181966.1	Kenya	29/11/2012
Orthopneumovirus	*Orthopneumovirus hominis*	Respiratory syncytial virus A	KJ643488.1	USA	2013
Orthopneumovirus	*Orthopneumovirus hominis*	Respiratory syncytial virus A	MH181972.1	Kenya	15/12/2012
Orthopneumovirus	*Orthopneumovirus hominis*	Respiratory syncytial virus A	MN630099.1	USA	19/1/2016
Orthopneumovirus	*Orthopneumovirus hominis*	Respiratory syncytial virus A	OR466334.1	USA	26/10/2012
Orthopneumovirus	*Orthopneumovirus hominis*	Respiratory syncytial virus A	OK649646.1	Brazil	2010
Orthopneumovirus	*Orthopneumovirus hominis*	Respiratory syncytial virus A	OK649616.1	Brazil	2008
Orthopneumovirus	*Orthopneumovirus hominis*	Respiratory syncytial virus A	OK649643.1	Brazil	2010
Orthopneumovirus	*Orthopneumovirus hominis*	Respiratory syncytial virus A	MH182035.1	Kenya	24/1/2015
Orthopneumovirus	*Orthopneumovirus hominis*	Respiratory syncytial virus A	PP882672.1	USA	18/10/2023
Orthopneumovirus	*Orthopneumovirus hominis*	Respiratory syncytial virus A	PP795136.1	USA	16/10/2023
Orthopneumovirus	*Orthopneumovirus hominis*	Respiratory syncytial virus A	PP970029.1	Ireland	29/10/2023
Orthopneumovirus	*Orthopneumovirus hominis*	Respiratory syncytial virus A	OQ024128.1	USA	3/11/2022
Orthopneumovirus	*Orthopneumovirus hominis*	Respiratory syncytial virus A	PP819400.1	China	14/1/2024
Orthopneumovirus	*Orthopneumovirus hominis*	Respiratory syncytial virus A	OQ171912.1	USA	12/7/2022
Orthopneumovirus	*Orthopneumovirus hominis*	Respiratory syncytial virus A	PP342422.1	USA	4/12/2023
Orthopneumovirus	*Orthopneumovirus hominis*	Respiratory syncytial virus A	PP352335.1	USA	1/10/2022
Orthopneumovirus	*Orthopneumovirus hominis*	Respiratory syncytial virus A	PP970013.1	Ireland	30/11/2022
Orthopneumovirus	*Orthopneumovirus hominis*	Respiratory syncytial virus A	PP681248.1	USA	6/12/2022

Orthopneumovirus	*Orthopneumovirus hominis*	Respiratory syncytial virus A	PP970032.1	Ireland	31/10/2023
Orthopneumovirus	*Orthopneumovirus hominis*	Respiratory syncytial virus A	PQ618043.1	Panama	22/7/2024
Orthopneumovirus	*Orthopneumovirus hominis*	Respiratory syncytial virus A	PP970033.1	Ireland	26/10/2023
Orthopneumovirus	*Orthopneumovirus hominis*	Respiratory syncytial virus A	OP890329.1	USA	1/11/2022
Orthopneumovirus	*Orthopneumovirus hominis*	Respiratory syncytial virus A	PP760404.1	USA	1/12/2022
Orthopneumovirus	*Orthopneumovirus hominis*	Respiratory syncytial virus A	PQ618025.1	Panama	3/10/2022
Orthopneumovirus	*Orthopneumovirus hominis*	Respiratory syncytial virus A	OR795472.1	Germany	14/10/2022
Orthopneumovirus	*Orthopneumovirus hominis*	Respiratory syncytial virus A	OQ024111.1	USA	8/11/2022
Orthopneumovirus	*Orthopneumovirus hominis*	Respiratory syncytial virus A	OP890340.1	USA	1/10/2022
Orthopneumovirus	*Orthopneumovirus hominis*	Respiratory syncytial virus A	PP978496.1	USA	2024
Orthopneumovirus	*Orthopneumovirus hominis*	Respiratory syncytial virus A	PP530261.1	USA	3/1/2024
Orthopneumovirus	*Orthopneumovirus hominis*	Respiratory syncytial virus A	PP352382.1	USA	1/11/2022
Orthopneumovirus	*Orthopneumovirus hominis*	Respiratory syncytial virus A	PQ618079.1	Panama	11/8/2023
Orthopneumovirus	*Orthopneumovirus hominis*	Respiratory syncytial virus A	OQ171901.1	USA	18/10/2022
Orthopneumovirus	*Orthopneumovirus hominis*	Respiratory syncytial virus A	PQ348881.1	UK	16/10/2022
Orthopneumovirus	*Orthopneumovirus hominis*	Respiratory syncytial virus A	PP882676.1	USA	21/11/2023
Orthopneumovirus	*Orthopneumovirus hominis*	Respiratory syncytial virus A	OR601477.1	USA	19/12/2022
Orthopneumovirus	*Orthopneumovirus hominis*	Respiratory syncytial virus A	PP970061.1	Ireland	19/10/2023
Orthopneumovirus	*Orthopneumovirus hominis*	Respiratory syncytial virus A	OQ024139.1	USA	4/11/2022
Orthopneumovirus	*Orthopneumovirus hominis*	Respiratory syncytial virus A	PP795151.1	USA	22/11/2023
Orthopneumovirus	*Orthopneumovirus hominis*	Respiratory syncytial virus A	PQ618026.1	Panama	22/9/2022
Orthopneumovirus	*Orthopneumovirus hominis*	Respiratory syncytial virus A	OQ024147.1	USA	3/11/2022
Orthopneumovirus	*Orthopneumovirus hominis*	Respiratory syncytial virus A	PQ610202.1	Argentina	11/5/2023
Orthopneumovirus	*Orthopneumovirus hominis*	Respiratory syncytial virus A	PP352337.1	USA	1/11/2022
Orthopneumovirus	*Orthopneumovirus hominis*	Respiratory syncytial virus A	PP352330.1	USA	1/10/2022
Orthopneumovirus	*Orthopneumovirus hominis*	Respiratory syncytial virus A	PP969960.1	Ireland	27/11/2023
Orthopneumovirus	*Orthopneumovirus hominis*	Respiratory syncytial virus A	OP890317.1	USA	2022
Orthopneumovirus	*Orthopneumovirus hominis*	Respiratory syncytial virus A	PQ349013.1	UK	4/8/2022
Orthopneumovirus	*Orthopneumovirus hominis*	Respiratory syncytial virus A	PP352346.1	USA	1/10/2022
Orthopneumovirus	*Orthopneumovirus hominis*	Respiratory syncytial virus A	PQ348879.1	UK	12/10/2022
Orthopneumovirus	*Orthopneumovirus hominis*	Respiratory syncytial virus A	PP411984.1	Thailand	12/8/2023

Orthopneumovirus	*Orthopneumovirus hominis*	Respiratory syncytial virus A	PP203262.1	USA	2022
Orthopneumovirus	*Orthopneumovirus hominis*	Respiratory syncytial virus A	PP681255.1	USA	8/12/2022
Orthopneumovirus	*Orthopneumovirus hominis*	Respiratory syncytial virus A	OQ024148.1	USA	3/11/2022
Orthopneumovirus	*Orthopneumovirus hominis*	Respiratory syncytial virus A	PQ348864.1	UK	14/8/2022
Orthopneumovirus	*Orthopneumovirus hominis*	Respiratory syncytial virus A	PP795132.1	USA	5/10/2023
Orthopneumovirus	*Orthopneumovirus hominis*	Respiratory syncytial virus A	PP969993.1	Ireland	29/11/2023
Orthopneumovirus	*Orthopneumovirus hominis*	Respiratory syncytial virus A	LC816571.1	Japan	8/8/2023
Orthopneumovirus	*Orthopneumovirus hominis*	Respiratory syncytial virus A	PP833560.1	China	1/3/2024
Orthopneumovirus	*Orthopneumovirus hominis*	Respiratory syncytial virus A	PQ610209.1	Argentina	15/5/2023
Orthopneumovirus	*Orthopneumovirus hominis*	Respiratory syncytial virus A	PP151366.1	Kuwait	18/9/2021
Orthopneumovirus	*Orthopneumovirus hominis*	Respiratory syncytial virus A	MZ515945.1	Netherlands	11/12/2018
Orthopneumovirus	*Orthopneumovirus hominis*	Respiratory syncytial virus A	OQ024145.1	USA	14/11/2022
Orthopneumovirus	*Orthopneumovirus hominis*	Respiratory syncytial virus A	ON237307.1	Argentina	22/5/2017
Orthopneumovirus	*Orthopneumovirus hominis*	Respiratory syncytial virus A	OR795378.1	Germany	13/2/2019
Orthopneumovirus	*Orthopneumovirus hominis*	Respiratory syncytial virus A	PP151342.1	Kuwait	16/9/2021
Orthopneumovirus	*Orthopneumovirus hominis*	Respiratory syncytial virus A	PQ618041.1	Panama	20/9/2022
Orthopneumovirus	*Orthopneumovirus hominis*	Respiratory syncytial virus A	PQ348865.1	UK	28/8/2022
Orthopneumovirus	*Orthopneumovirus hominis*	Respiratory syncytial virus A	PP525317.1	USA	2016
Orthopneumovirus	*Orthopneumovirus hominis*	Respiratory syncytial virus A	PP681270.1	USA	20/12/2022
Orthopneumovirus	*Orthopneumovirus hominis*	Respiratory syncytial virus A	PP376612.1	Japan	14/11/2019
Orthopneumovirus	*Orthopneumovirus hominis*	Respiratory syncytial virus A	PP495874.1	USA	19/12/2019
Orthopneumovirus	*Orthopneumovirus hominis*	Respiratory syncytial virus A	PP151390.1	Kuwait	4/9/2021
Orthopneumovirus	*Orthopneumovirus hominis*	Respiratory syncytial virus A	PP151386.1	Kuwait	9/9/2021
Orthopneumovirus	*Orthopneumovirus hominis*	Respiratory syncytial virus A	OR287930.1	USA	5/1/2020
Orthopneumovirus	*Orthopneumovirus hominis*	Respiratory syncytial virus A	PP495914.1	USA	8/1/2020
Orthopneumovirus	*Orthopneumovirus hominis*	Respiratory syncytial virus A	OQ171906.1	USA	27/7/2022
Orthopneumovirus	*Orthopneumovirus hominis*	Respiratory syncytial virus A	PP376331.1	Canada	15/12/2019
Orthopneumovirus	*Orthopneumovirus hominis*	Respiratory syncytial virus A	PP795139.1	USA	26/10/2023
Orthopneumovirus	*Orthopneumovirus hominis*	Respiratory syncytial virus A	PP376698.1	Canada	21/11/2019
Orthopneumovirus	*Orthopneumovirus hominis*	Respiratory syncytial virus A	OQ024144.1	USA	3/11/2022
Orthopneumovirus	*Orthopneumovirus hominis*	Respiratory syncytial virus A	PQ618063.1	Panama	8/8/2019

Orthopneumovirus	*Orthopneumovirus hominis*	Respiratory syncytial virus A	PP376790.1	Netherlands	4/1/2018
Orthopneumovirus	*Orthopneumovirus hominis*	Respiratory syncytial virus A	MZ515951.1	UK	14/11/2018
Orthopneumovirus	*Orthopneumovirus hominis*	Respiratory syncytial virus A	MZ515718.1	Netherlands	20/12/2019
Orthopneumovirus	*Orthopneumovirus hominis*	Respiratory syncytial virus A	PP376655.1	Netherlands	18/12/2019
Orthopneumovirus	*Orthopneumovirus hominis*	Respiratory syncytial virus A	MZ515632.1	Netherlands	3/12/2018
Orthopneumovirus	*Orthopneumovirus hominis*	Respiratory syncytial virus A	PP376519.1	Australia	5/11/2020
Orthopneumovirus	*Orthopneumovirus hominis*	Respiratory syncytial virus A	PP681274.1	USA	3/1/2023
Orthopneumovirus	*Orthopneumovirus hominis*	Respiratory syncytial virus A	PP376388.1	Netherlands	20/12/2018
Orthopneumovirus	*Orthopneumovirus hominis*	Respiratory syncytial virus A	PP376456.1	South Africa	27/2/2018
Orthopneumovirus	*Orthopneumovirus hominis*	Respiratory syncytial virus A	OM857356.1	Australia	7/2/2021
Orthopneumovirus	*Orthopneumovirus hominis*	Respiratory syncytial virus A	MZ515842.1	Spain	10/1/2020
Orthopneumovirus	*Orthopneumovirus hominis*	Respiratory syncytial virus A	OP890319.1	USA	1/11/2022
Orthopneumovirus	*Orthopneumovirus hominis*	Respiratory syncytial virus A	PP376682.1	Japan	2/9/2019
Orthopneumovirus	*Orthopneumovirus hominis*	Respiratory syncytial virus A	PP376837.1	UK	9/12/2019
Orthopneumovirus	*Orthopneumovirus hominis*	Respiratory syncytial virus A	MZ516044.1	Netherlands	27/11/2019
Orthopneumovirus	*Orthopneumovirus hominis*	Respiratory syncytial virus A	PP376539.1	Netherlands	7/12/2018
Orthopneumovirus	*Orthopneumovirus hominis*	Respiratory syncytial virus A	OR666560.1	China	3/2/2020
Orthopneumovirus	*Orthopneumovirus hominis*	Respiratory syncytial virus A	PQ618074.1	Panama	27/6/2024
Orthopneumovirus	*Orthopneumovirus hominis*	Respiratory syncytial virus A	OR287986.1	USA	18/8/2021
Orthopneumovirus	*Orthopneumovirus hominis*	Respiratory syncytial virus A	MZ516080.1	Netherlands	21/11/2019
Orthopneumovirus	*Orthopneumovirus hominis*	Respiratory syncytial virus A	PP376341.1	Canada	17/1/2019
Orthopneumovirus	*Orthopneumovirus hominis*	Respiratory syncytial virus A	MZ515647.1	UK	20/11/2018
Orthopneumovirus	*Orthopneumovirus hominis*	Respiratory syncytial virus A	PP376636.1	South Africa	16/3/2018
Orthopneumovirus	*Orthopneumovirus hominis*	Respiratory syncytial virus A	OM857340.1	Australia	12/1/2021
Orthopneumovirus	*Orthopneumovirus hominis*	Respiratory syncytial virus A	OR795406.1	Germany	29/2/2020
Orthopneumovirus	*Orthopneumovirus hominis*	Respiratory syncytial virus A	PP376429.1	Australia	25/1/2021
Orthopneumovirus	*Orthopneumovirus hominis*	Respiratory syncytial virus A	PP376667.1	France	19/12/2018
Género	Especie	Nombre del virus	Número de acceso	País	Fecha
Orthopneumovirus	*Orthopneumovirus hominis*	Respiratory syncytial virus B	OQ941661.1	Australia	1977
Orthopneumovirus	*Orthopneumovirus hominis*	Respiratory syncytial virus B	OQ941662.1	Australia	1977
Orthopneumovirus	*Orthopneumovirus hominis*	Respiratory syncytial virus B	MG642045.1	USA	1979

Orthopneumovirus	*Orthopneumovirus hominis*	Respiratory syncytial virus B	MG642037.1	USA	1980
Orthopneumovirus	*Orthopneumovirus hominis*	Respiratory syncytial virus B	MG642047.1	USA	1980
Orthopneumovirus	*Orthopneumovirus hominis*	Respiratory syncytial virus B	MG642065.1	USA	1981
Orthopneumovirus	*Orthopneumovirus hominis*	Respiratory syncytial virus B	MG642025.1	USA	1982
Orthopneumovirus	*Orthopneumovirus hominis*	Respiratory syncytial virus B	MG642043.1	USA	1982
Orthopneumovirus	*Orthopneumovirus hominis*	Respiratory syncytial virus B	MG642044.1	USA	1982
Orthopneumovirus	*Orthopneumovirus hominis*	Respiratory syncytial virus B	MG642078.1	USA	1982
Orthopneumovirus	*Orthopneumovirus hominis*	Respiratory syncytial virus B	MG642059.1	USA	1983
Orthopneumovirus	*Orthopneumovirus hominis*	Respiratory syncytial virus B	MG642046.1	USA	1984
Orthopneumovirus	*Orthopneumovirus hominis*	Respiratory syncytial virus B	MG642057.1	USA	1984
Orthopneumovirus	*Orthopneumovirus hominis*	Respiratory syncytial virus B	MG642039.1	USA	1985
Orthopneumovirus	*Orthopneumovirus hominis*	Respiratory syncytial virus B	MG642042.1	USA	1985
Orthopneumovirus	*Orthopneumovirus hominis*	Respiratory syncytial virus B	MG642051.1	USA	1986
Orthopneumovirus	*Orthopneumovirus hominis*	Respiratory syncytial virus B	MG642068.1	USA	1987
Orthopneumovirus	*Orthopneumovirus hominis*	Respiratory syncytial virus B	MG642076.1	USA	1987
Orthopneumovirus	*Orthopneumovirus hominis*	Respiratory syncytial virus B	MG642036.1	USA	1989
Orthopneumovirus	*Orthopneumovirus hominis*	Respiratory syncytial virus B	MG642082.1	USA	1989
Orthopneumovirus	*Orthopneumovirus hominis*	Respiratory syncytial virus B	OK649745.1	USA	1990
Orthopneumovirus	*Orthopneumovirus hominis*	Respiratory syncytial virus B	MG642066.1	USA	1990
Orthopneumovirus	*Orthopneumovirus hominis*	Respiratory syncytial virus B	OK649749.1	USA	1991
Orthopneumovirus	*Orthopneumovirus hominis*	Respiratory syncytial virus B	MG642027.1	USA	1991
Orthopneumovirus	*Orthopneumovirus hominis*	Respiratory syncytial virus B	MG642054.1	USA	1991
Orthopneumovirus	*Orthopneumovirus hominis*	Respiratory syncytial virus B	MG642072.1	USA	1991
Orthopneumovirus	*Orthopneumovirus hominis*	Respiratory syncytial virus B	OK649747.1	USA	1992
Orthopneumovirus	*Orthopneumovirus hominis*	Respiratory syncytial virus B	OK649748.1	USA	1992
Orthopneumovirus	*Orthopneumovirus hominis*	Respiratory syncytial virus B	PQ416568.1	USA	1993
Orthopneumovirus	*Orthopneumovirus hominis*	Respiratory syncytial virus B	PQ416569.1	USA	1993
Orthopneumovirus	*Orthopneumovirus hominis*	Respiratory syncytial virus B	PQ416570.1	USA	1993
Orthopneumovirus	*Orthopneumovirus hominis*	Respiratory syncytial virus B	MG642049.1	USA	1993
Orthopneumovirus	*Orthopneumovirus hominis*	Respiratory syncytial virus B	OK649750.1	USA	1994
Orthopneumovirus	*Orthopneumovirus hominis*	Respiratory syncytial virus B	OK649751.1	USA	1994

Orthopneumovirus	*Orthopneumovirus hominis*	Respiratory syncytial virus B	OK649752.1	USA	1994
Orthopneumovirus	*Orthopneumovirus hominis*	Respiratory syncytial virus B	OK649740.1	Canada	1995
Orthopneumovirus	*Orthopneumovirus hominis*	Respiratory syncytial virus B	OK649746.1	USA	1995
Orthopneumovirus	*Orthopneumovirus hominis*	Respiratory syncytial virus B	MG642064.1	USA	1995
Orthopneumovirus	*Orthopneumovirus hominis*	Respiratory syncytial virus B	PQ591399.1	UK	1996
Orthopneumovirus	*Orthopneumovirus hominis*	Respiratory syncytial virus B	MG642062.1	USA	1996
Orthopneumovirus	*Orthopneumovirus hominis*	Respiratory syncytial virus B	MZ568943.1	Germany	20/12/2010
Orthopneumovirus	*Orthopneumovirus hominis*	Respiratory syncytial virus B	MH594423.1	Kenya	1/3/2010
Orthopneumovirus	*Orthopneumovirus hominis*	Respiratory syncytial virus B	MZ568992.1	Germany	11/2/2011
Orthopneumovirus	*Orthopneumovirus hominis*	Respiratory syncytial virus B	MH594422.1	Kenya	6/3/2010
Orthopneumovirus	*Orthopneumovirus hominis*	Respiratory syncytial virus B	KY249669.1	UK	6/12/2012
Orthopneumovirus	*Orthopneumovirus hominis*	Respiratory syncytial virus B	MZ568977.1	Germany	23/2/2007
Orthopneumovirus	*Orthopneumovirus hominis*	Respiratory syncytial virus B	MH594391.1	Kenya	5/4/2010
Orthopneumovirus	*Orthopneumovirus hominis*	Respiratory syncytial virus B	KY249670.1	UK	4/12/2012
Orthopneumovirus	*Orthopneumovirus hominis*	Respiratory syncytial virus B	MZ568975.1	Germany	27/1/2009
Orthopneumovirus	*Orthopneumovirus hominis*	Respiratory syncytial virus B	OK649697.1	Brazil	2009
Orthopneumovirus	*Orthopneumovirus hominis*	Respiratory syncytial virus B	OK649739.1	Brazil	2010
Orthopneumovirus	*Orthopneumovirus hominis*	Respiratory syncytial virus B	KY249667.1	UK	21/12/2012
Orthopneumovirus	*Orthopneumovirus hominis*	Respiratory syncytial virus B	MZ568952.1	Germany	20/1/2005
Orthopneumovirus	*Orthopneumovirus hominis*	Respiratory syncytial virus B	PQ591398.1	UK	1997
Orthopneumovirus	*Orthopneumovirus hominis*	Respiratory syncytial virus B	OQ848551.1	USA	1/1/2011
Orthopneumovirus	*Orthopneumovirus hominis*	Respiratory syncytial virus B	MZ568939.1	Germany	7/12/2010
Orthopneumovirus	*Orthopneumovirus hominis*	Respiratory syncytial virus B	MZ568987.1	Germany	26/3/2012
Orthopneumovirus	*Orthopneumovirus hominis*	Respiratory syncytial virus B	MZ568938.1	Germany	8/12/2003
Orthopneumovirus	*Orthopneumovirus hominis*	Respiratory syncytial virus B	OK649742.1	Kenya	2007
Orthopneumovirus	*Orthopneumovirus hominis*	Respiratory syncytial virus B	KY249680.1	UK	1/12/2011
Orthopneumovirus	*Orthopneumovirus hominis*	Respiratory syncytial virus B	PQ416567.1	USA	2004
Orthopneumovirus	*Orthopneumovirus hominis*	Respiratory syncytial virus B	MZ568946.1	Germany	6/1/2005
Orthopneumovirus	*Orthopneumovirus hominis*	Respiratory syncytial virus B	OK649688.1	Brazil	2009
Orthopneumovirus	*Orthopneumovirus hominis*	Respiratory syncytial virus B	OK649713.1	Brazil	2009
Orthopneumovirus	*Orthopneumovirus hominis*	Respiratory syncytial virus B	MZ568958.1	Germany	15/1/2011

Orthopneumovirus	*Orthopneumovirus hominis*	Respiratory syncytial virus B	OK649709.1	Brazil	2009
Orthopneumovirus	*Orthopneumovirus hominis*	Respiratory syncytial virus B	OK649727.1	Brazil	2010
Orthopneumovirus	*Orthopneumovirus hominis*	Respiratory syncytial virus B	OR466400.1	USA	24/2/2012
Orthopneumovirus	*Orthopneumovirus hominis*	Respiratory syncytial virus B	OK649744.1	Kenya	2009
Orthopneumovirus	*Orthopneumovirus hominis*	Respiratory syncytial virus B	MZ568982.1	Germany	1/4/2004
Orthopneumovirus	*Orthopneumovirus hominis*	Respiratory syncytial virus B	OK649734.1	Brazil	2010
Orthopneumovirus	*Orthopneumovirus hominis*	Respiratory syncytial virus B	MH594397.1	Kenya	8/4/2010
Orthopneumovirus	*Orthopneumovirus hominis*	Respiratory syncytial virus B	OK649735.1	Brazil	2010
Orthopneumovirus	*Orthopneumovirus hominis*	Respiratory syncytial virus B	KY249679.1	UK	22/12/2011
Orthopneumovirus	*Orthopneumovirus hominis*	Respiratory syncytial virus B	MH594390.1	Kenya	2/4/2010
Orthopneumovirus	*Orthopneumovirus hominis*	Respiratory syncytial virus B	OK649732.1	Brazil	2010
Orthopneumovirus	*Orthopneumovirus hominis*	Respiratory syncytial virus B	OK649698.1	Brazil	2009
Orthopneumovirus	*Orthopneumovirus hominis*	Respiratory syncytial virus B	KY249668.1	UK	6/12/2012
Orthopneumovirus	*Orthopneumovirus hominis*	Respiratory syncytial virus B	MH594437.1	Kenya	5/4/2010
Orthopneumovirus	*Orthopneumovirus hominis*	Respiratory syncytial virus B	MH594410.1	Kenya	23/2/2010
Orthopneumovirus	*Orthopneumovirus hominis*	Respiratory syncytial virus B	MK109789.1	Jordan	10/3/2011
Orthopneumovirus	*Orthopneumovirus hominis*	Respiratory syncytial virus B	MH594402.1	Kenya	27/1/2010
Orthopneumovirus	*Orthopneumovirus hominis*	Respiratory syncytial virus B	MK109770.1	Jordan	29/4/2010
Orthopneumovirus	*Orthopneumovirus hominis*	Respiratory syncytial virus B	OK649741.1	Kenya	2007
Orthopneumovirus	*Orthopneumovirus hominis*	Respiratory syncytial virus B	OK649716.1	Brazil	2009
Orthopneumovirus	*Orthopneumovirus hominis*	Respiratory syncytial virus B	MH594421.1	Kenya	8/3/2010
Orthopneumovirus	*Orthopneumovirus hominis*	Respiratory syncytial virus B	ON237208.1	Argentina	18/7/2016
Orthopneumovirus	*Orthopneumovirus hominis*	Respiratory syncytial virus B	PP377468.1	Spain	7/12/2017
Orthopneumovirus	*Orthopneumovirus hominis*	Respiratory syncytial virus B	OK649718.1	Brazil	2009
Orthopneumovirus	*Orthopneumovirus hominis*	Respiratory syncytial virus B	ON237092.1	Argentina	21/5/2014
Orthopneumovirus	*Orthopneumovirus hominis*	Respiratory syncytial virus B	PP377304.1	Netherlands	20/12/2017
Orthopneumovirus	*Orthopneumovirus hominis*	Respiratory syncytial virus B	KY249677.1	UK	6/1/2012
Orthopneumovirus	*Orthopneumovirus hominis*	Respiratory syncytial virus B	MF001044.1	USA	2015
Orthopneumovirus	*Orthopneumovirus hominis*	Respiratory syncytial virus B	LC385000.1	Philippines	12/10/2015
Orthopneumovirus	*Orthopneumovirus hominis*	Respiratory syncytial virus B	ON237169.1	Argentina	28/3/2016
Orthopneumovirus	*Orthopneumovirus hominis*	Respiratory syncytial virus B	KY249656.1	UK	7/1/2014

Orthopneumovirus	*Orthopneumovirus hominis*	Respiratory syncytial virus B	KY249682.1	UK	10/1/2016
Orthopneumovirus	*Orthopneumovirus hominis*	Respiratory syncytial virus B	LC384997.1	Philippines	30/7/2014
Orthopneumovirus	*Orthopneumovirus hominis*	Respiratory syncytial virus B	MK109778.1	Jordan	20/12/2012
Orthopneumovirus	*Orthopneumovirus hominis*	Respiratory syncytial virus B	OR466377.1	USA	15/12/2014
Orthopneumovirus	*Orthopneumovirus hominis*	Respiratory syncytial virus B	MK749880.1	Nicaragua	14/10/2015
Orthopneumovirus	*Orthopneumovirus hominis*	Respiratory syncytial virus B	MZ568970.1	Germany	26/1/2015
Orthopneumovirus	*Orthopneumovirus hominis*	Respiratory syncytial virus B	MH594441.1	Kenya	25/3/2010
Orthopneumovirus	*Orthopneumovirus hominis*	Respiratory syncytial virus B	OQ848548.1	USA	1/1/2011
Orthopneumovirus	*Orthopneumovirus hominis*	Respiratory syncytial virus B	OK649714.1	Brazil	2009
Orthopneumovirus	*Orthopneumovirus hominis*	Respiratory syncytial virus B	MK749868.1	Nicaragua	1/9/2016
Orthopneumovirus	*Orthopneumovirus hominis*	Respiratory syncytial virus B	PP377222.1	Spain	15/11/2017
Orthopneumovirus	*Orthopneumovirus hominis*	Respiratory syncytial virus B	MK749873.1	Nicaragua	20/11/2015
Orthopneumovirus	*Orthopneumovirus hominis*	Respiratory syncytial virus B	PP376994.1	Netherlands	22/12/2017
Orthopneumovirus	*Orthopneumovirus hominis*	Respiratory syncytial virus B	PP974173.1	China	18/12/2016
Orthopneumovirus	*Orthopneumovirus hominis*	Respiratory syncytial virus B	PP974178.1	China	20/12/2016
Orthopneumovirus	*Orthopneumovirus hominis*	Respiratory syncytial virus B	OK649703.1	Brazil	2009
Orthopneumovirus	*Orthopneumovirus hominis*	Respiratory syncytial virus B	MK749915.1	Nicaragua	7/1/2016
Orthopneumovirus	*Orthopneumovirus hominis*	Respiratory syncytial virus B	ON237141.1	Argentina	2/6/2015
Orthopneumovirus	*Orthopneumovirus hominis*	Respiratory syncytial virus B	MK749889.1	Nicaragua	11/11/2015
Orthopneumovirus	*Orthopneumovirus hominis*	Respiratory syncytial virus B	ON237130.1	Argentina	4/5/2015
Orthopneumovirus	*Orthopneumovirus hominis*	Respiratory syncytial virus B	ON237087.1	Argentina	14/5/2014
Orthopneumovirus	*Orthopneumovirus hominis*	Respiratory syncytial virus B	MK109769.1	Jordan	2/5/2011
Orthopneumovirus	*Orthopneumovirus hominis*	Respiratory syncytial virus B	ON237106.1	Argentina	10/6/2014
Orthopneumovirus	*Orthopneumovirus hominis*	Respiratory syncytial virus B	ON237164.1	Argentina	20/7/2015
Orthopneumovirus	*Orthopneumovirus hominis*	Respiratory syncytial virus B	MK109782.1	Jordan	15/1/2013
Orthopneumovirus	*Orthopneumovirus hominis*	Respiratory syncytial virus B	OQ848541.1	USA	1/11/2010
Orthopneumovirus	*Orthopneumovirus hominis*	Respiratory syncytial virus B	PP376910.1	Netherlands	16/11/2017
Orthopneumovirus	*Orthopneumovirus hominis*	Respiratory syncytial virus B	PP376938.1	Netherlands	18/12/2017
Orthopneumovirus	*Orthopneumovirus hominis*	Respiratory syncytial virus B	OR466387.1	USA	23/1/2015
Orthopneumovirus	*Orthopneumovirus hominis*	Respiratory syncytial virus B	PP377377.1	Finland	27/12/2017
Orthopneumovirus	*Orthopneumovirus hominis*	Respiratory syncytial virus B	MH594418.1	Kenya	19/2/2010

Orthopneumovirus	*Orthopneumovirus hominis*	Respiratory syncytial virus B	ON237138.1	Argentina	27/5/2015
Orthopneumovirus	*Orthopneumovirus hominis*	Respiratory syncytial virus B	MZ515743.1	Netherlands	28/12/2017
Orthopneumovirus	*Orthopneumovirus hominis*	Respiratory syncytial virus B	MK109788.1	Jordan	18/2/2013
Orthopneumovirus	*Orthopneumovirus hominis*	Respiratory syncytial virus B	MK749898.1	Nicaragua	23/12/2015
Orthopneumovirus	*Orthopneumovirus hominis*	Respiratory syncytial virus B	ON237202.1	Argentina	5/7/2016
Orthopneumovirus	*Orthopneumovirus hominis*	Respiratory syncytial virus B	ON237146.1	Argentina	8/6/2015
Orthopneumovirus	*Orthopneumovirus hominis*	Respiratory syncytial virus B	ON237160.1	Argentina	7/7/2015
Orthopneumovirus	*Orthopneumovirus hominis*	Respiratory syncytial virus B	MZ515710.1	Netherlands	8/12/2017
Orthopneumovirus	*Orthopneumovirus hominis*	Respiratory syncytial virus B	PP377354.1	Mexico	25/2/2020
Orthopneumovirus	*Orthopneumovirus hominis*	Respiratory syncytial virus B	PP377291.1	Spain	2/12/2017
Orthopneumovirus	*Orthopneumovirus hominis*	Respiratory syncytial virus B	PP377346.1	South_Africa	24/6/2019
Orthopneumovirus	*Orthopneumovirus hominis*	Respiratory syncytial virus B	PP377020.1	South_Africa	24/5/2019
Orthopneumovirus	*Orthopneumovirus hominis*	Respiratory syncytial virus B	PP828957.1	China	22/2/2024
Orthopneumovirus	*Orthopneumovirus hominis*	Respiratory syncytial virus B	PP377305.1	France	20/12/2018
Orthopneumovirus	*Orthopneumovirus hominis*	Respiratory syncytial virus B	OR795265.1	Germany	25/10/2022
Orthopneumovirus	*Orthopneumovirus hominis*	Respiratory syncytial virus B	PP970083.1	Ireland	16/11/2022
Orthopneumovirus	*Orthopneumovirus hominis*	Respiratory syncytial virus B	PP377252.1	South_Africa	17/4/2018
Orthopneumovirus	*Orthopneumovirus hominis*	Respiratory syncytial virus B	PP377249.1	Russia	17/3/2020
Orthopneumovirus	*Orthopneumovirus hominis*	Respiratory syncytial virus B	OR795242.1	Germany	18/10/2021
Orthopneumovirus	*Orthopneumovirus hominis*	Respiratory syncytial virus B	OM857375.1	Australia	6/3/2020
Orthopneumovirus	*Orthopneumovirus hominis*	Respiratory syncytial virus B	OM857390.1	Australia	1/2/2020
Orthopneumovirus	*Orthopneumovirus hominis*	Respiratory syncytial virus B	PP342499.1	USA	17/12/2023
Orthopneumovirus	*Orthopneumovirus hominis*	Respiratory syncytial virus B	MZ515824.1	UK	23/11/2018
Orthopneumovirus	*Orthopneumovirus hominis*	Respiratory syncytial virus B	PP871358.1	USA	28/10/2023
Orthopneumovirus	*Orthopneumovirus hominis*	Respiratory syncytial virus B	PP376847.1	Netherlands	10/12/2019
Orthopneumovirus	*Orthopneumovirus hominis*	Respiratory syncytial virus B	OR795254.1	Germany	15/11/2021
Orthopneumovirus	*Orthopneumovirus hominis*	Respiratory syncytial virus B	PP377191.1	South_Africa	12/3/2018
Orthopneumovirus	*Orthopneumovirus hominis*	Respiratory syncytial virus B	PP377163.1	Russia	10/3/2020
Orthopneumovirus	*Orthopneumovirus hominis*	Respiratory syncytial virus B	PP770455.1	USA	16/1/2024
Orthopneumovirus	*Orthopneumovirus hominis*	Respiratory syncytial virus B	PP377214.1	Germany	14/2/2019
Orthopneumovirus	*Orthopneumovirus hominis*	Respiratory syncytial virus B	MZ515975.1	Netherlands	14/12/2019

Orthopneumovirus	*Orthopneumovirus hominis*	Respiratory syncytial virus B	PP377230.1	Japan	15/7/2018
Orthopneumovirus	*Orthopneumovirus hominis*	Respiratory syncytial virus B	PP376995.1	Finland	22/2/2018
Orthopneumovirus	*Orthopneumovirus hominis*	Respiratory syncytial virus B	MZ516041.1	Netherlands	18/12/2018
Orthopneumovirus	*Orthopneumovirus hominis*	Respiratory syncytial virus B	MZ569010.1	Germany	20/4/2018
Orthopneumovirus	*Orthopneumovirus hominis*	Respiratory syncytial virus B	OR326732.1	USA	29/10/2019
Orthopneumovirus	*Orthopneumovirus hominis*	Respiratory syncytial virus B	PP377455.1	South_Korea	6/1/2020
Orthopneumovirus	*Orthopneumovirus hominis*	Respiratory syncytial virus B	PP084063.1	USA	22/10/2023
Orthopneumovirus	*Orthopneumovirus hominis*	Respiratory syncytial virus B	PP495998.1	USA	25/12/2019
Orthopneumovirus	*Orthopneumovirus hominis*	Respiratory syncytial virus B	MZ515683.1	Netherlands	5/1/2018
Orthopneumovirus	*Orthopneumovirus hominis*	Respiratory syncytial virus B	MZ515625.1	Netherlands	30/1/2018
Orthopneumovirus	*Orthopneumovirus hominis*	Respiratory syncytial virus B	PP377389.1	Russia	28/1/2020
Orthopneumovirus	*Orthopneumovirus hominis*	Respiratory syncytial virus B	PP377110.1	Australia	4/5/2019
Orthopneumovirus	*Orthopneumovirus hominis*	Respiratory syncytial virus B	PP377425.1	Netherlands	31/12/2017
Orthopneumovirus	*Orthopneumovirus hominis*	Respiratory syncytial virus B	PP377102.1	Netherlands	4/1/2019
Orthopneumovirus	*Orthopneumovirus hominis*	Respiratory syncytial virus B	PP377288.1	Spain	2/1/2018
Orthopneumovirus	*Orthopneumovirus hominis*	Respiratory syncytial virus B	PP377282.1	South_Africa	19/3/2018
Orthopneumovirus	*Orthopneumovirus hominis*	Respiratory syncytial virus B	MZ515978.1	UK	26/11/2018
Orthopneumovirus	*Orthopneumovirus hominis*	Respiratory syncytial virus B	PP377053.1	South_Korea	27/2/2020
Orthopneumovirus	*Orthopneumovirus hominis*	Respiratory syncytial virus B	LC816574.1	Japan	20/9/2022
Orthopneumovirus	*Orthopneumovirus hominis*	Respiratory syncytial virus B	PP377071.1	Canada	29/3/2019
Orthopneumovirus	*Orthopneumovirus hominis*	Respiratory syncytial virus B	PP377004.1	South_Africa	22/7/2019
Orthopneumovirus	*Orthopneumovirus hominis*	Respiratory syncytial virus B	PP377030.1	South_Africa	25/4/2019
Orthopneumovirus	*Orthopneumovirus hominis*	Respiratory syncytial virus B	PP377382.1	Mexico	27/2/2020
Orthopneumovirus	*Orthopneumovirus hominis*	Respiratory syncytial virus B	PP882673.1	USA	31/10/2023
Orthopneumovirus	*Orthopneumovirus hominis*	Respiratory syncytial virus B	PP342478.1	USA	10/12/2023
Orthopneumovirus	*Orthopneumovirus hominis*	Respiratory syncytial virus B	PP970080.1	Ireland	8/12/2022
Orthopneumovirus	*Orthopneumovirus hominis*	Respiratory syncytial virus B	MT373703.1	Russia	14/3/2019

Table S4. RV-A, RV-B and RV-C reference sequences with collection date and geographic origin used for temporal analyses. This table presents the reference RV sequences included in the evolutionary and time-scaled phylogenetic analyses. For each sequence the viral genus, species, virus name, GenBank accession number, country of origin and year of collection are presented.

Genus	Specie	Genotype	Accession Number	Country	Collection Date
Enterovirus	*Enterovirus alpharhino*	RV-1A	KC894167.1	USA	2011
Enterovirus	*Enterovirus alpharhino*	RV-A	GQ223229.1	China	19/7/2007
Enterovirus	*Enterovirus alpharhino*	RV-A	MW587078.1	China	1/12/2017
Enterovirus	*Enterovirus alpharhino*	RV-A	PP158741.1	USA	13/4/2021
Enterovirus	*Enterovirus alpharhino*	RV-A	MW587065.1	China	1/12/2017
Enterovirus	*Enterovirus alpharhino*	RV-A	KR871677.1	China	17/6/2013
Enterovirus	*Enterovirus alpharhino*	RV-A	MH899591.1	USA	1/1/2015
Enterovirus	*Enterovirus alpharhino*	RV-A	MW587090.1	China	1/12/2017
Enterovirus	*Enterovirus alpharhino*	RV-A	MW587079.1	China	1/12/2017
Enterovirus	*Enterovirus alpharhino*	RV-A	JQ837718.1	USA	2000
Enterovirus	*Enterovirus alpharhino*	RV-A	JQ837716.1	USA	2000
Enterovirus	*Enterovirus alpharhino*	RV-A101	OM001440.1	USA	2021
Enterovirus	*Enterovirus alpharhino*	RV-A101	MZ363438.1	USA	10/5/2021
Enterovirus	*Enterovirus alpharhino*	RV-A101	MZ835593.1	USA	2021
Enterovirus	*Enterovirus alpharhino*	RV-A101	MZ629112.1	USA	27/5/2021
Enterovirus	*Enterovirus alpharhino*	RV-A101	MZ363468.1	USA	2021
Enterovirus	*Enterovirus alpharhino*	RV-A101	OK539466.1	USA	14/7/2021
Enterovirus	*Enterovirus alpharhino*	RV-A101	ON311204.1	USA	7/4/2021
Enterovirus	*Enterovirus alpharhino*	RV-A101	MZ835568.1	USA	2021
Enterovirus	*Enterovirus alpharhino*	RV-A101	MZ629156.1	USA	1/6/2021
Enterovirus	*Enterovirus alpharhino*	RV-A101	MZ629126.1	USA	5/6/2021
Enterovirus	*Enterovirus alpharhino*	RV-A101	MZ629176.1	USA	1/6/2021
Enterovirus	*Enterovirus alpharhino*	RV-A101	OM001398.1	USA	21/6/2021
Enterovirus	*Enterovirus alpharhino*	RV-A101	MZ460708.1	USA	14/5/2021
Enterovirus	*Enterovirus alpharhino*	RV-A103	MH517019.1	India	2018
Enterovirus	*Enterovirus alpharhino*	RV-A105	MZ268661.1	USA	26/4/2021
Enterovirus	*Enterovirus alpharhino*	RV-A11	MZ670589.1	USA	9/6/2021

Enterovirus	*Enterovirus alpharhino*	RV-A1B	OL365069.1	USA	15/6/2021
Enterovirus	*Enterovirus alpharhino*	RV-A1B	OL365071.1	USA	15/6/2021
Enterovirus	*Enterovirus alpharhino*	RV-A1B	OL638404.1	USA	10/7/2021
Enterovirus	*Enterovirus alpharhino*	RV-A1B	OM001345.1	USA	22/6/2021
Enterovirus	*Enterovirus alpharhino*	RV-A1B	OM001347.1	USA	22/6/2021
Enterovirus	*Enterovirus alpharhino*	RV-A1B	OM001357.1	USA	9/7/2021
Enterovirus	*Enterovirus alpharhino*	RV-A1B	OM001364.1	USA	9/7/2021
Enterovirus	*Enterovirus alpharhino*	RV-A1B	OM001369.1	USA	2/7/2021
Enterovirus	*Enterovirus alpharhino*	RV-A1B	OM001370.1	USA	3/7/2021
Enterovirus	*Enterovirus alpharhino*	RV-A1B	OM001371.1	USA	1/7/2021
Enterovirus	*Enterovirus alpharhino*	RV-A1B	OK649414.1	USA	18/6/2021
Enterovirus	*Enterovirus alpharhino*	RV-A1B	OM001410.1	USA	21/6/2021
Enterovirus	*Enterovirus alpharhino*	RV-A1B	MZ835589.1	USA	10/6/2021
Enterovirus	*Enterovirus alpharhino*	RV-A1B	OK649390.1	USA	2021
Enterovirus	*Enterovirus alpharhino*	RV-A1B	MZ670573.1	USA	9/6/2021
Enterovirus	*Enterovirus alpharhino*	RV-A1B	OK649401.1	USA	2021
Enterovirus	*Enterovirus alpharhino*	RV-A1B	OL638414.1	USA	2021
Enterovirus	*Enterovirus alpharhino*	RV-A1B	OK181470.1	USA	2021
Enterovirus	*Enterovirus alpharhino*	RV-A1B	OK161357.1	USA	8/6/2021
Enterovirus	*Enterovirus alpharhino*	RV-A1B	OK181477.1	USA	2021
Enterovirus	*Enterovirus alpharhino*	RV-A1B	OK539463.1	USA	2021
Enterovirus	*Enterovirus alpharhino*	RV-A1B	OK181453.1	USA	21/6/2021
Enterovirus	*Enterovirus alpharhino*	RV-A1B	OK539483.1	USA	2/7/2021
Enterovirus	*Enterovirus alpharhino*	RV-A1B	OL638430.1	USA	2021
Enterovirus	*Enterovirus alpharhino*	RV-A1B	OK539461.1	USA	10/7/2021
Enterovirus	*Enterovirus alpharhino*	RV-A1B	OL638443.1	USA	2021
Enterovirus	*Enterovirus alpharhino*	RV-A1B	OK161365.1	USA	8/6/2021
Enterovirus	*Enterovirus alpharhino*	RV-A1B	OL133749.1	USA	2021
Enterovirus	*Enterovirus alpharhino*	RV-A20	OK181488.1	USA	21/6/2021
Enterovirus	*Enterovirus alpharhino*	RV-A20	MZ363466.1	USA	8/5/2021
Enterovirus	*Enterovirus alpharhino*	RV-A21	LC699421.1	Japan	2020

Enterovirus	*Enterovirus alpharhino*	RV-A22	OP342736.1	China	30/3/2017
Enterovirus	*Enterovirus alpharhino*	RV-A22	OM001446.1	USA	22/6/2021
Enterovirus	*Enterovirus alpharhino*	RV-A22	OK539478.1	USA	14/7/2021
Enterovirus	*Enterovirus alpharhino*	RV-A22	MZ629173.1	USA	7/6/2021
Enterovirus	*Enterovirus alpharhino*	RV-A22	KY369885.1	USA	2016
Enterovirus	*Enterovirus alpharhino*	RV-A22	ON311254.1	USA	21/4/2021
Enterovirus	*Enterovirus alpharhino*	RV-A23	ON311235.1	USA	9/9/2020
Enterovirus	*Enterovirus alpharhino*	RV-A23	MZ835617.1	USA	2021
Enterovirus	*Enterovirus alpharhino*	RV-A23	ON311229.1	USA	14/9/2020
Enterovirus	*Enterovirus alpharhino*	RV-A23	MZ629158.1	USA	5/6/2021
Enterovirus	*Enterovirus alpharhino*	RV-A23	OK017935.1	USA	25/5/2021
Enterovirus	*Enterovirus alpharhino*	RV-A23	ON311231.1	USA	9/9/2020
Enterovirus	*Enterovirus alpharhino*	RV-A23	ON311239.1	USA	25/8/2020
Enterovirus	*Enterovirus alpharhino*	RV-A23	OK649369.1	USA	28/6/2021
Enterovirus	*Enterovirus alpharhino*	RV-A23	ON311250.1	USA	10/8/2020
Enterovirus	*Enterovirus alpharhino*	RV-A23	ON311232.1	USA	9/9/2020
Enterovirus	*Enterovirus alpharhino*	RV-A24	OK254868.1	USA	2021
Enterovirus	*Enterovirus alpharhino*	RV-A24	OK649393.1	USA	2021
Enterovirus	*Enterovirus alpharhino*	RV-A25	MZ363461.1	USA	27/4/2021
Enterovirus	*Enterovirus alpharhino*	RV-A30	OP342733.1	China	28/12/2017
Enterovirus	*Enterovirus alpharhino*	RV-A30	OL638454.1	USA	16/6/2021
Enterovirus	*Enterovirus alpharhino*	RV-A30	OM001394.1	USA	2/7/2021
Enterovirus	*Enterovirus alpharhino*	RV-A30	OM001444.1	USA	1/7/2021
Enterovirus	*Enterovirus alpharhino*	RV-A30	OM001453.1	USA	2021
Enterovirus	*Enterovirus alpharhino*	RV-A30	OM001459.1	USA	2021
Enterovirus	*Enterovirus alpharhino*	RV-A30	OK017961.1	USA	27/5/2021
Enterovirus	*Enterovirus alpharhino*	RV-A30	OK161359.1	USA	8/6/2021
Enterovirus	*Enterovirus alpharhino*	RV-A30	OK161360.1	USA	8/6/2021
Enterovirus	*Enterovirus alpharhino*	RV-A30	OK254828.1	USA	28/6/2021
Enterovirus	*Enterovirus alpharhino*	RV-A32	FJ445127.1	NA	1959
Enterovirus	*Enterovirus alpharhino*	RV-A34	OM001368.1	USA	9/7/2021

Enterovirus	*Enterovirus alpharhino*	RV-A36	DQ473505.1	USA	2006
Enterovirus	*Enterovirus alpharhino*	RV-A39	OK649372.1	USA	28/6/2021
Enterovirus	*Enterovirus alpharhino*	RV-A39	OL638420.1	USA	10/7/2021
Enterovirus	*Enterovirus alpharhino*	RV-A39	OL638442.1	USA	2021
Enterovirus	*Enterovirus alpharhino*	RV-A39	MZ629175.1	USA	1/6/2021
Enterovirus	*Enterovirus alpharhino*	RV-A40	LC720413.1	Japan	2020
Enterovirus	*Enterovirus alpharhino*	RV-A45	OP342727.1	China	12/2/2018
Enterovirus	*Enterovirus alpharhino*	RV-A49	OL133734.1	USA	15/7/2021
Enterovirus	*Enterovirus alpharhino*	RV-A49	OL133741.1	USA	10/7/2021
Enterovirus	*Enterovirus alpharhino*	RV-A53	OP342724.1	China	30/1/2018
Enterovirus	*Enterovirus alpharhino*	RV-A53	OK017946.1	USA	27/5/2021
Enterovirus	*Enterovirus alpharhino*	RV-A53	MN306049.1	USA	2019
Enterovirus	*Enterovirus alpharhino*	RV-A54	OK539505.1	USA	14/7/2021
Enterovirus	*Enterovirus alpharhino*	RV-A54	OP342722.1	China	31/1/2018
Enterovirus	*Enterovirus alpharhino*	RV-A56	OP342719.1	China	10/6/2014
Enterovirus	*Enterovirus alpharhino*	RV-A58	LC699420.1	Japan	2019
Enterovirus	*Enterovirus alpharhino*	RV-A58	MZ667420.1	USA	2019
Enterovirus	*Enterovirus alpharhino*	RV-A61	KY369886.1	USA	2016
Enterovirus	*Enterovirus alpharhino*	RV-A61	KY189313.1	USA	2016
Enterovirus	*Enterovirus alpharhino*	RV-A66	OK649378.1	USA	16/6/2021
Enterovirus	*Enterovirus alpharhino*	RV-A66	OK161406.1	USA	15/6/2021
Enterovirus	*Enterovirus alpharhino*	RV-A66	MZ835579.1	USA	7/6/2021
Enterovirus	*Enterovirus alpharhino*	RV-A66	MN749158.1	USA	2015
Enterovirus	*Enterovirus alpharhino*	RV-A66	JN112340.1	USA	5/3/2008
Enterovirus	*Enterovirus alpharhino*	RV-A66	OK539487.1	USA	2/7/2021
Enterovirus	*Enterovirus alpharhino*	RV-A67	ON311214.1	USA	7/11/2020
Enterovirus	*Enterovirus alpharhino*	RV-A68	OP342715.1	China	7/5/2014
Enterovirus	*Enterovirus alpharhino*	RV-A7	ON311264.1	USA	26/3/2021
Enterovirus	*Enterovirus alpharhino*	RV-A75	DQ473510.1	USA	2006
Enterovirus	*Enterovirus alpharhino*	RV-A77	PP411922.1	Thailand	12/7/2023
Enterovirus	*Enterovirus alpharhino*	RV-A78	OK181500.1	USA	18/6/2021

Enterovirus	*Enterovirus alpharhino*	RV-A78	OK649418.1	USA	28/6/2021
Enterovirus	*Enterovirus alpharhino*	RV-A78	OM001434.1	USA	2021
Enterovirus	*Enterovirus alpharhino*	RV-A78	OK254871.1	USA	21/6/2021
Enterovirus	*Enterovirus alpharhino*	RV-A78	OL638448.1	USA	2021
Enterovirus	*Enterovirus alpharhino*	RV-A78	OK254855.1	USA	28/6/2021
Enterovirus	*Enterovirus alpharhino*	RV-A78	OM001386.1	USA	6/7/2021
Enterovirus	*Enterovirus alpharhino*	RV-A80	LC720412.1	Japan	2021
Enterovirus	*Enterovirus alpharhino*	RV-A81	LC699417.1	Japan	2019
Enterovirus	*Enterovirus alpharhino*	RV-A9	OP342742.1	China	16/5/2014
Enterovirus	*Enterovirus betarhino*	RV-B	JF285331.1	Switzerland	30/12/2004
Enterovirus	*Enterovirus betarhino*	RV-B100	MZ629178.1	USA	7/6/2021
Enterovirus	*Enterovirus betarhino*	RV-B100	MZ153262.1	USA	15/4/2021
Enterovirus	*Enterovirus betarhino*	RV-B27	OK539488.1	USA	2/7/2021
Enterovirus	*Enterovirus betarhino*	RV-B35	ON311181.1	USA	16/3/2020
Enterovirus	*Enterovirus betarhino*	RV-B48	JN990698.1	USA	2008
Enterovirus	*Enterovirus betarhino*	RV-B6	OM001402.1	USA	2021
Enterovirus	*Enterovirus betarhino*	RV-B6	MZ835566.1	USA	4/6/2021
Enterovirus	*Enterovirus betarhino*	RV-B6	MZ835615.1	USA	7/6/2021
Enterovirus	*Enterovirus betarhino*	RV-B6	OL133766.1	USA	15/7/2021
Enterovirus	*Enterovirus betarhino*	RV-B6	OK181464.1	USA	18/6/2021
Enterovirus	*Enterovirus betarhino*	RV-B6	MZ363443.1	USA	1/5/2021
Enterovirus	*Enterovirus betarhino*	RV-B70	OL961525.1	USA	12/5/2021
Enterovirus	*Enterovirus betarhino*	RV-B70	OM001422.1	USA	2021
Enterovirus	*Enterovirus betarhino*	RV-B70	MK501735.1	Colombia	2018
Enterovirus	*Enterovirus betarhino*	RV-B91	MZ268667.1	USA	1/5/2021
Enterovirus	*Enterovirus betarhino*	RV-B91	MZ153267.1	USA	9/4/2021
Enterovirus	*Enterovirus betarhino*	RV-B91	KX494871.1	China	25/12/2016
Enterovirus	*Enterovirus betarhino*	RV-B91	OL961542.1	USA	12/5/2021
Enterovirus	*Enterovirus cerhino*	RV-C	KY189320.1	USA	2016
Enterovirus	*Enterovirus cerhino*	RV-C	MW587060.1	China	1/12/2017
Enterovirus	*Enterovirus cerhino*	RV-C	MZ667421.2	USA	24/8/2009

Enterovirus	*Enterovirus cerhino*	RV-C	MN369033.1	USA	2018
Enterovirus	*Enterovirus cerhino*	RV-C	JF317014.1	China	1/12/2008
Enterovirus	*Enterovirus cerhino*	RV-C11	OQ348028.1	USA	1/11/2022
Enterovirus	*Enterovirus cerhino*	RV-C11	OM001344.1	USA	2021
Enterovirus	*Enterovirus cerhino*	RV-C11	OM001465.1	USA	21/6/2021
Enterovirus	*Enterovirus cerhino*	RV-C11	OK254853.1	USA	21/6/2021
Enterovirus	*Enterovirus cerhino*	RV-C11	OK649407.1	USA	2021
Enterovirus	*Enterovirus cerhino*	RV-C11	OK649422.1	USA	16/6/2021
Enterovirus	*Enterovirus cerhino*	RV-C11	OK017910.1	USA	2021
Enterovirus	*Enterovirus cerhino*	RV-C11	MW969520.1	USA	23/2/2021
Enterovirus	*Enterovirus cerhino*	RV-C11	MN163127.1	China	1/3/2017
Enterovirus	*Enterovirus cerhino*	RV-C11	EU840952.2	Switzerland	1/4/2008
Enterovirus	*Enterovirus cerhino*	RV-C11	MZ363429.1	USA	19/4/2021
Enterovirus	*Enterovirus cerhino*	RV-C11	MZ322922.1	USA	7/4/2021
Enterovirus	*Enterovirus cerhino*	RV-C11	MZ322928.1	USA	7/4/2021
Enterovirus	*Enterovirus cerhino*	RV-C11	MW969518.1	USA	22/2/2021
Enterovirus	*Enterovirus cerhino*	RV-C11	MZ268710.1	USA	3/5/2021
Enterovirus	*Enterovirus cerhino*	RV-C15	OP342697.1	China	6/2/2018
Enterovirus	*Enterovirus cerhino*	RV-C15	OP342698.1	China	26/12/2017
Enterovirus	*Enterovirus cerhino*	RV-C15	OP342700.1	China	19/5/2014
Enterovirus	*Enterovirus cerhino*	RV-C15	ON311169.1	USA	19/1/2021
Enterovirus	*Enterovirus cerhino*	RV-C15	ON311170.1	USA	16/1/2021
Enterovirus	*Enterovirus cerhino*	RV-C15	ON311171.1	USA	11/1/2021
Enterovirus	*Enterovirus cerhino*	RV-C15	ON311172.1	USA	11/1/2021
Enterovirus	*Enterovirus cerhino*	RV-C15	GU219984.1	USA	4/4/2007
Enterovirus	*Enterovirus cerhino*	RV-C15	MZ153254.1	USA	13/4/2021
Enterovirus	*Enterovirus cerhino*	RV-C15	ON311173.1	USA	16/11/2020
Enterovirus	*Enterovirus cerhino*	RV-C17	OR726585.1	USA	1/11/2020
Enterovirus	*Enterovirus cerhino*	RV-C17	OR726586.1	USA	1/11/2020
Enterovirus	*Enterovirus cerhino*	RV-C17	OQ116583.1	USA	2019
Enterovirus	*Enterovirus cerhino*	RV-C17	OL365070.1	USA	28/6/2021

Enterovirus	*Enterovirus cerhino*	RV-C17	OL961526.1	USA	6/7/2021
Enterovirus	*Enterovirus cerhino*	RV-C17	OK017945.1	USA	25/5/2021
Enterovirus	*Enterovirus cerhino*	RV-C17	OK017955.1	USA	27/5/2021
Enterovirus	*Enterovirus cerhino*	RV-C17	OK161409.1	USA	15/6/2021
Enterovirus	*Enterovirus cerhino*	RV-C17	OK181457.1	USA	17/6/2021
Enterovirus	*Enterovirus cerhino*	RV-C17	MZ670590.1	USA	9/6/2021
Enterovirus	*Enterovirus cerhino*	RV-C17	MZ629181.1	USA	5/6/2021
Enterovirus	*Enterovirus cerhino*	RV-C17	MZ268699.1	USA	13/4/2021
Enterovirus	*Enterovirus cerhino*	RV-C17	MZ629165.1	USA	1/6/2021
Enterovirus	*Enterovirus cerhino*	RV-C17	MZ460700.1	USA	14/5/2021
Enterovirus	*Enterovirus cerhino*	RV-C17	MZ268721.1	USA	21/4/2021
Enterovirus	*Enterovirus cerhino*	RV-C2	ON881132.1	China	2/8/2018
Enterovirus	*Enterovirus cerhino*	RV-C2	ON881130.1	China	19/11/2018
Enterovirus	*Enterovirus cerhino*	RV-C20	MW969519.1	USA	22/2/2021
Enterovirus	*Enterovirus cerhino*	RV-C26	OQ331223.1	USA	1/3/2020
Enterovirus	*Enterovirus cerhino*	RV-C3	OK181493.1	USA	21/6/2021
Enterovirus	*Enterovirus cerhino*	RV-C3	MN228693.1	USA	3/2/2019
Enterovirus	*Enterovirus cerhino*	RV-C3	MZ153256.1	USA	13/4/2021
Enterovirus	*Enterovirus cerhino*	RV-C3	OK017931.1	USA	25/5/2021
Enterovirus	*Enterovirus cerhino*	RV-C3	OM001455.1	USA	2021
Enterovirus	*Enterovirus cerhino*	RV-C3	OK161371.1	USA	14/6/2021
Enterovirus	*Enterovirus cerhino*	RV-C3	MZ268662.1	USA	2021
Enterovirus	*Enterovirus cerhino*	RV-C3	MZ153270.1	USA	9/4/2021
Enterovirus	*Enterovirus cerhino*	RV-C33	MZ670597.1	USA	1/6/2021
Enterovirus	*Enterovirus cerhino*	RV-C36	MZ835600.1	USA	2021
Enterovirus	*Enterovirus cerhino*	RV-C36	MZ221147.1	USA	19/4/2021
Enterovirus	*Enterovirus cerhino*	RV-C36	OM001437.1	USA	2021
Enterovirus	*Enterovirus cerhino*	RV-C40	OK254858.1	USA	29/6/2021
Enterovirus	*Enterovirus cerhino*	RV-C40	MZ268694.1	USA	2021
Enterovirus	*Enterovirus cerhino*	RV-C40	MZ363459.1	USA	6/5/2021
Enterovirus	*Enterovirus cerhino*	RV-C40	MW969535.1	USA	22/2/2021

Enterovirus	*Enterovirus cerhino*	RV-C42	MH752985.1	USA	26/1/2016
Enterovirus	*Enterovirus cerhino*	RV-C42	OK181472.1	USA	18/6/2021
Enterovirus	*Enterovirus cerhino*	RV-C42	MZ460705.1	USA	12/5/2021
Enterovirus	*Enterovirus cerhino*	RV-C44	MZ629168.1	USA	1/6/2021
Enterovirus	*Enterovirus cerhino*	RV-C54	KP282614.1	Spain	11/5/2009
Enterovirus	*Enterovirus cerhino*	RV-C56	MW969529.1	USA	22/2/2021
Enterovirus	*Enterovirus cerhino*	RV-C56	MZ322916.1	USA	26/4/2021
Enterovirus	*Enterovirus cerhino*	RV-C56	MZ629123.1	USA	1/6/2021
